# Barriers and facilitators to implementing workplace interventions to promote mental health: qualitative evidence synthesis

**DOI:** 10.1186/s13643-024-02569-2

**Published:** 2024-06-07

**Authors:** Charlotte Paterson, Caleb Leduc, Margaret Maxwell, Birgit Aust, Heather Strachan, Ainslie O’Connor, Fotini Tsantila, Johanna Cresswell-Smith, Gyorgy Purebl, Lars Winter, Naim Fanaj, Asmae Doukani, Bridget Hogg, Paul Corcoran, Luigia D’Alessandro, Sharna Mathieu, Ulrich Hegerl, Ella Arensman, Birgit A. Greiner, The MENTUPP Consortium, Andia Meksi, Andras Szekely, Ariel Como, Arilda Dushaj, Arlinda Cerga, Azucena Justicia, Benedikt Amann, Chantal Van Audenhove, Chris Lockwood, Cliodhna O’Connor, Doireann Ni Dhalaigh, Dooyoung Kim, Eileen Williamson, Eva Zsak, Eve Griffin, Evelien Coppens, Genc Burazeri, Gentiana Qirjako, Grace Davey, Hanna Reich de Paredes, Jaap Van Weeghel, Juan Carlos Medina Alcaraz, Juliane Hug, Kahar Abula, Kairi Kõlves, Karen Mulcahy, Katherine Thomson, Kristian Wahlbeck, Laura Cox, Mallorie Leduc, Marta Fontana McNally, Pia Hauck, Reiner Rugulies, Ruth Benson, Saara Rapeli, Sarita Sanches, Sevim Mustafa, Stefan Hackel, Tanya King, Vanda Scott, Víctor Pérez Solà, Victoria Ross, Wendy Orchard

**Affiliations:** 1 Nursing, Midwifery and Allied Health Professional Research Unit, University of Stirling, Pathfoot Building, Stirling, FK9 4LA Scotland, UK; 2 School of Public Health, University College Cork, Western Gateway Building, Cork, Ireland; 3 National Suicide Research Foundation, Western Gateway Building, Cork, Ireland; 4 National Research Centre for the Working Environment, Lersø Parkallé 105, Copenhagen, 2100 Denmark; 5 Pintail Limited, Co., Dublin, Ireland; 6 LUCAS, Centre for Care Research and Consultancy, KU Leuven, Louvain, 3000 Belgium; 7 Finnish Institute for Health and Welfare (THL) Equality Unit–Mental Health Team, Helsinki, Finland; 8 Institute of Behavioural Sciences, Semmelweis University, Budapest, Hungary; 9 Phrenos Center of Expertise for Severe Mental Illnesses, Utrecht, the Netherlands; 10 Mental Health Center Prizren, Prizren, Kosovo; 11 Almae Mater Europaea Campus College Rezonanca, Prishtina, Kosovo; 12 Centre for Global Mental Health, Department of Population Health, London School of Hygiene & Tropical Medicine, Keppel Street, London, WC1E 7HT UK; 13 Centre Fòrum Research Unit, Institute of Mental Health, Hospital del Mar Barcelona, Barcelona, SpainHospital del Mar Research Institute, Barcelona, Spain; 14 Centro de Investigación Biomédica en Red de Salud Mental (CIBERSAM), Instituto Carlos III, Madrid, Spain; 15 International Association for Suicide Prevention (IASP), 5221 Wisconsin Avenue NW, Washington, DC, 20015 USA; 16 Australian Institute for Suicide Research and Prevention, World Health Organization Collaborating Centre for Research and Training in Suicide Prevention, Brisbane, Australia; 17 School of Applied Psychology, Griffith University, Mt. Gravatt Campus, Brisbane, QLD 4122 Australia; 18 European Alliance Against Depression E.V., Leipzig, 04109 Germany; 19 Department of Psychiatry, Psychosomatic Medicine and Psychotherapy, University Hospital, Goethe University, Frankfurt Am Main, 60528 Germany; 20 Australian Institute for Suicide Research and Prevention, School of Applied Psychology, Griffith University, Brisbane, Australia

**Keywords:** Barriers and facilitators, Workplace, Mental health, Implementation science, Scoping review, Systematic review, Organisation interventions, Wellbeing

## Abstract

**Background:**

Despite growing interest in workplace mental health interventions, evidence of their effectiveness is mixed. Implementation science offers a valuable lens to investigate the factors influencing successful implementation. However, evidence synthesis is lacking, especially for small-to-medium-sized enterprises (SMEs) and for specific work sectors. The objectives of this review are to establish the scope of research with explicit analysis of implementation aspects of workplace mental health interventions and to identify barriers and facilitators to implementation in general and within SMEs and selected sectors.

**Methods:**

A systematic scoping review and meta-synthesis of mixed methods process evaluation research from 11 databases, with the evaluation of methodological quality (MMAT) and confidence in findings (CERQual), was conducted. We selected information-rich studies and synthesised them using domains within the Nielsen and Randall implementation framework: context, intervention activities, implementation; and mental models.

**Results:**

We included 43 studies published between 2009 and 2022, of which 22 were rated as information-rich to be analysed for barriers and facilitators. Most studies were conducted in healthcare. Facilitators reflecting ‘high confidence’ included: relevant and tailored content, continuous and pro-active leadership buy-in and support, internal or external change agents/champions, assistance from managers and peers, resources, and senior-level experience and awareness of mental health issues. Healthcare sector-specific facilitators included: easy accessibility with time provided, fostering relationships, clear communication, and perceptions of the intervention. Stigma and confidentiality issues were reported as barriers overall. Due to the small number of studies within SMEs reported findings did not reach ‘high confidence’. A lack of studies in construction and Information and Communication Technology meant separate analyses were not possible.

**Conclusions:**

There is dependable evidence of key factors for the implementation of workplace mental health interventions which should be used to improve implementation. However, there is a lack of studies in SMEs and in a larger variety of sectors.

**Systematic review registration:**

Research Registry (reviewregistry897).

**Supplementary Information:**

The online version contains supplementary material available at 10.1186/s13643-024-02569-2.

## Background

### What is the problem?

Mental health and well-being are vital concerns to hundreds of millions of working people worldwide. The World Health Organisation (WHO) estimated that 15% of working-age adults experience a mental disorder at any point in time [1]. This increased during the COVID-19 pandemic with an estimated 25% rise in the prevalence of anxiety and depression worldwide in 2020 [2, 3]. The issue extends beyond individual disease burden and affects the productivity, competitiveness, and sustainability of private and public organisations due to sickness absence and presenteeism [4–6]. Lost productivity due to depression and anxiety is estimated to cost the global economy 1 trillion US dollars [7]. Subsequently, the protection and promotion of workplace mental health has increasingly gained attention in many organisations and was highlighted by recent ‘WHO Guidelines on Mental Health at Work’ [7, 8].

### Workplace mental health interventions

Two main types of workplace mental health interventions have evolved [9]. *Worker-directed* interventions, also called individual intervention approaches, aim to enhance the individual worker’s knowledge, skills, awareness, and competencies to cope with stressful working conditions, and support to seek help, when facing mental health challenges (e.g. mindfulness training). *Work-directed* approaches, also called organisational intervention approaches, aim to improve psychosocial working conditions and the organisation of work relevant to mental health and wellbeing (e.g. flexible working hours). Integrated mental health intervention models suggest a combination of work-directed and worker-directed strategies for maximum population health gain [9, 10], with multi-level approaches [11], i.e. interventions addressing several or all levels of the IGLOO model (individual-group-leaders-organisation-outer context) [12].

### The search for explanations of failed and successful interventions

While the effectiveness of workplace mental health interventions has been documented for a range of outcomes [13–18], the evidence is not entirely consistent [19–21]. Research into specific mechanisms and process factors associated with the successful delivery of mental health interventions in the workplace is limited, which led several authors to call for more attention to these aspects. For example, Burgess et al. identified the need for thorough process evaluation to reduce what they called a ‘trial and error approach’ [22]. By that, they mean the lack of a theoretical framework for why an intervention is expected to lead to a specific outcome so that findings can be integrated with and built upon existing research. Without that, the authors argue, failures just lead to trying again with a somewhat different approach but without a deeper understanding of the barriers that might be in the way of positive outcomes.

Also, previous calls for more differentiation between intervention and theory failure [23] are still relevant. We need to understand better if the lack of the expected outcomes of an intervention was due to shortcomings in the way the intervention was implemented or if the underlying theory about how the intervention would work was wrong. As the number of workplace interventions that do not reach the expected outcomes continues to be high, scholars have called for more specific implementation and evaluation research [24–26]. Implementation science can provide a useful lens for examining the ‘how’ and ‘why’ interventions work or fail. An overview of failed interventions revealed reasons related to the intervention, context, and process [27]. Synthesised evidence of reasons for unsuccessful or successful implementation of workplace interventions is also likely to have some relevance for workplace mental health interventions [21–23, 27–29]. Workplace mental health intervention may however face specific challenges for implementation due to stigma and discrimination attached to mental health [9], warranting a separate synthesis of implementation barriers and facilitators. There has been a growing body of process evaluations linked to workplace mental health interventions, which made it possible to conduct a systematic review of 74 qualitative and quantitative process evaluation studies on implementation practices in workplace health and psychological well-being interventions [30]. The review revealed three key success factors: a continued effort to the intervention and its adaptation; functional learning structures; and consultative governance structures. A meta-synthesis of qualitative research provided a detailed description of barriers and facilitators for the implementation of workplace mental health interventions but was limited to studies published from 2019 to 2021 and not well supported by the evidence based on a quality rating. Findings for facilitators comprised line manager support, completion of intervention activities during working hours, scheduling flexibility, and trainer credibility. Barriers included high workload and understaffing, lack of priority given to the intervention by managers and lack of appropriate facilitator training [26].

Not covered in existing evidence synthesis is the context-specificity of implementation by the industrial sector or occupation, with implementation factors likely to vary by sector. For example, there are specific challenges for implementing mental health interventions in the construction industry, with hindrances caused by, e.g. frequently changing work sites, long working hours, and a culture with traditional masculine values such as self-reliance and stoicism, which limit help-seeking behaviour [31–33]. The Information and Communication Technology (ICT) sector has rapidly expanded due to the ongoing digital transformation [34] and also faces specific challenges for the implementation of mental health interventions. The ICT work environment has been characterised as chaotic, turbulent, and constantly changing, requiring workers to work long hours with expectations to remain constantly available online [35, 36]. There is also evidence that frequent mergers and organisational change interfere with the implementation of mental health interventions [37]. In comparison, most process evaluation studies have been conducted in the healthcare setting, although there has been less attention to SMEs within the healthcare sector [26].

Furthermore, there is little evidence (of barriers and facilitators) for the implementation of interventions in small-to-medium-sized enterprises (SMEs) which are less likely to implement health promotion programmes than larger companies [38]. Among the barriers that are being reported for SME participation in workplace health interventions are lack of interest, lack of support by management and concerns about privacy [39, 40]. SMEs may face further challenges such as business owners experiencing substantial responsibility for implementation, high workloads, and psychological stress due to limited resources and capacity, recruitment, and retention issues [41–43].

### Aim and research questions

The general aim of this review is to collate and critically appraise workplace mental health intervention implementation literature to understand how and why some interventions are more effectively implemented than others. This review is part of the international MENTUPP project (Mental Health Promotion and Intervention in Occupational Settings, www.mentupp.eu) [11, 44]. The review aims to provide evidence-based guidance for the MENTUPP project and future projects for the implementation of multi-level interventions to improve mental health and well-being with a particular focus on SMEs in three sectors with high prevalence rates of mental health problems, namely, information and communication technology (ICT), healthcare, and construction sectors. To provide the best possible support for the objectives of the MENTUPP project we focussed on the following research questions (RQs):

- RQ1. What is the scope of research with an explicit focus on implementation aspects of mental health promotion interventions in the workplace?
- RQ2. What are the barriers and facilitators to implementing mental health promotion interventions in the workplace?
- RQ3. What are the barriers and facilitators to implementing mental health promotion interventions in healthcare, ICT, construction, (RQ3a), and SMEs (RQ3b)?

## Methods

We conducted a scoping review and qualitative evidence synthesis (QES) to address the review aims, following guidance from Arksey et al. [45], Levac et al. [46], the Preferred Reporting Items for Systematic Reviews and Meta-Analyses (PRISMA) extension for scoping reviews (PRISMA-ScR) [47], Enhancing transparency in reporting the synthesis of qualitative research (ENTREQ) [48], and the Effective Practice and Organisation of Care (EPOC) QES [49] (see PRISMA and ENTREQ checklists in Additional file 1). The protocol was registered in the Research Registry (reviewregistry897) and subsequently published [50]. Differences between the protocol and review are reported in Additional file 2.

### Study designs

We included all study designs, which explicitly investigated or reported, in the title or abstract, any aspect of the implementation of mental health promotion interventions delivered in the workplace. We defined implementation as the delivery of an intervention in either the feasibility/pilot, evaluation, or implementation stage of the Medical Research Council (MRC) framework [51, 52]. We defined barriers and facilitators as any variable or condition that impedes or facilitates, respectively, the implementation of mental health promotion interventions.

We included literature reviews and primary research studies published either in peer-reviewed or grey literature. We excluded opinion pieces, commentaries, website discussions, blogs, magazines, newspaper articles, and books or chapters not reporting original research.

Studies published in English were included in step one of the search methods. Steps 2 and 3 included studies published in English, French, and German.

Studies published from April 2009 to August 2022 were included. Implementation science is a fairly new field of study and The WHO Global Plan of Action on Worker’s Health (2008–2017) [53] and the Mental Health Action Plan (2013–2030) [54] highlight the importance of promoting good mental health in the workplace, therefore, studies published from 2009 were deemed most relevant to this review.

### Outcomes of interest

Outcomes of interest included any implementation evaluation outcome scoring 3 or more on a data richness scale (see Table 1). We excluded studies only assessing the impact of interventions, i.e. evaluations of effectiveness/efficiency but not implementation.

**Table 1:** Adapted data richness scale

Score	Measure	Example
1	Very little qualitative data was presented that relates to the synthesis objective. Those findings that are presented are fairly descriptive and/or quantitative data that provided in-depth knowledge relating to the synthesis objective	A mixed-methods study using open-ended survey questions or a more detailed qualitative study where only part of the data relates to the synthesis objective
2	Some qualitative data presented that relate to the synthesis objective and/or quantitative data that provided in-depth knowledge relating to the synthesis objective	A limited number of qualitative findings from a mixed methods or qualitative study
3	A reasonable amount of qualitative data that relates to the synthesis objective and/or quantitative data that provides in-depth knowledge relating to the synthesis objective	A typical mixed methods or qualitative research journal publication with a smaller word limit using simple thematic analysis
4	A good amount and depth of qualitative data that relate to the synthesis objective	A qualitative research article in a journal that includes more context and setting descriptions and a more in-depth presentation of the findings
5	A large amount and depth of qualitative data that relate in depth to the synthesis objective	A detailed ethnography or a published qualitative article with the same objectives as the synthesis

Adapted from Ames et al. [55] and Ames et al. [56]

### Population

We included studies with participants (aged 16–65) in paid employment, including those on sick leave and who are returning to work. We excluded studies where the population was trainees, those in the armed forces, and those on sick leave.

### Setting

We included studies conducted in any geographical location that were set in the workplace. We defined workplace settings as any organisation operating with paid employees. Interventions must have been delivered through, or be associated with, the workplace and be implemented in the work schedule, work systems, or administrative structures. Sector-specific definitions from the European Commission were used [57]. The ICT sector included telecommunications activities, information technology activities and other information service activities (Div.61–63); the healthcare sector included healthcare provided by medical professionals in hospitals or other facilities and residential activities, but not social work activities (Div.86–87); and the construction sector included construction of buildings, civil engineering, and specialised construction activities (Div.41–43).

### Intervention types and targeted outcomes

We included interventions that aim to treat, prevent, or promote mental health [58]. Examples of included interventions are described in Table 2.

**Table 2:** Types of included interventions [9]

Category of intervention aim	Examples
To help protect mental health by reducing work-related risk factors	Job strain, poor working conditions, and job stressors such as job insecurity, psychological harassment (e.g. due to stigma), low social support at work, organisational injustice, and effort-reward imbalance
To promote workplace mental health well-being by creating positive aspects of work and developing employees’ strengths	satisfaction, well-being, psychological capital, positive mental health, resilience, and positive organisational attributes such as authentic leadership, supportive workplace culture, and workplace social capital
To respond to mental health problems when they occur	Interventions targeting individuals with mental health problems, such as burnout, stress, anxiety, depression or return to work for individuals with absence due to mental health problems

We excluded mental health interventions not specifically associated with workplace factors, or interventions not targeted for work contexts, not formally implemented in the workplace, and one-off events. We excluded studies with an explicit focus on addressing the impact of COVID-19 on staff well-being and mental health. Interventions not directly targeting mental health and mental well-being were included if the primary intervention outcome was related to mental health or mental well-being.

### Search strategy

We used iterative methods to develop and apply a comprehensive search strategy. To identify relevant studies, we combined free text terms and Medical Subject Headings for key concepts: (a) workplace AND (b) mental health AND (c) interventions AND (d) implementation. Where appropriate, Boolean operators and ‘wildcards’ were used. Where possible, we used an age filter for adults. A preliminary search strategy was developed for PsycINFO, using established search terms used in previous Cochrane and other reviews [59–61], and peer-reviewed in accordance with PRESS guidelines [62]. We adapted this strategy for each information source (see Additional file 3).

### Information sources

We used a stepwise approach to, first, identify reviews and map these against our review objectives [63]. Where gaps in evidence existed, we searched for primary studies and grey literature. Information sources were searched between April 2020 and August 2022 and are outlined below.

1. Scopus, PROSPERO, Health Technology Assessments, PubMed, Campbell Collaboration, Joanna Briggs Library, Web of Science Core Collection.
2. PsycINFO, Scopus, Pubmed, Web of Science Core Collection, CINAHL,
3. We conducted the following supplementary searches:
  - We conducted a grey literature search in the Institution of Occupational Safety and Health (IOSH) research.
  - Reference searching: relevant studies included in relevant systematic reviews [22, 64–66].
  - Google Scholar (25 pages relevant).
  - Personal contact: 14 international experts and authors of papers reporting evaluations of workplace interventions addressing mental health promotion, and seven of these responded [15, 33].

### Study selection

Three reviewers (CP, CL, HS) screened titles and abstracts for eligibility in Rayyan [67], rating them as relevant, irrelevant, or unsure. 15% of titles and abstracts were screened independently by all reviewers. Studies rated as irrelevant were immediately excluded. Full texts of the remaining studies were assessed independently by two reviewers (CP, CL, HS) against the selection criteria. Disagreements were resolved through discussion with a third reviewer (MM, BA, BAG).

### Study sampling

We developed and applied a stepped framework [56] to sample studies for RQ2 and RQ3. Each study was independently assessed by two reviewers (CP, CL, HS) for data richness using the scale in Table 1. To answer RQ2, we excluded studies that did not meet the criteria for data richness, i.e. scoring ≥ 4 on a data richness scale (see Table 1).

To answer RQ3, we included studies set in ICT, healthcare, and construction sectors, and in SMEs, scoring ≥ 3 on the data richness scale to account for potentially fewer articles (see Table 2). Studies including multiple sectors, were sampled if they reported separately on relevant sectors. Where studies did not report sector or organisation size, authors were contacted. We piloted the sampling framework on the first 10 studies. Discrepancies were discussed by the research team.

### Data extraction, synthesis, and presentation

We brought together multiple reports of the same study at data extraction and considered all publications related to that study, however, we only extracted quotes regarding barriers and facilitators to implementation from reports assessing implementation.

Multiple reviewers (CP, HS, CL, AO, AD, JCS, FT, BH, LDW, SM) systematically extracted data and used bespoke data extraction sheets in Microsoft Excel (Additional file 4). The extraction sheet was piloted on the first five studies and amended as required. Two reviewers independently extracted data from 15% of studies. All extracted data was cross-checked by a second reviewer (CP). Any disagreements were resolved through discussion with a third reviewer (CL, MM).

To address RQ1, we mapped all eligible primary research studies by extracting details of study characteristics (e.g. aim, design (coded according to EPOC [68]), setting (e.g. country, sector, organisation size (coded as small < 49 employees, medium 50–249 employees, or large > 250 employees [69], data richness (see Table 1), and country (coded using the World Bank) [70].

**Fig. 1:**
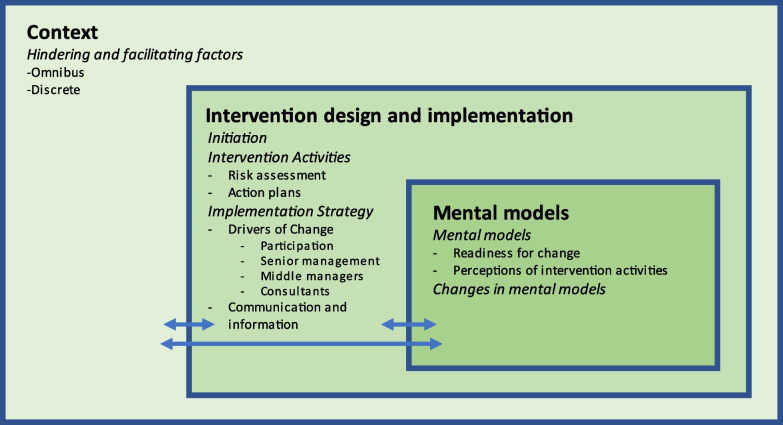
Nielsen and Randall’s [73] framework of factors influencing the implementation of occupational health interventions

To address RQ2, we extracted additional details of study design, intervention characteristics (guided by the TIDieR checklist) [71], and quotes providing rich data on barriers and facilitators to intervention implementation which were reported in the methods or results sections of studies. In the first stage of our synthesis, quotes were extracted verbatim and coded deductively using a best-fit framework [72]. Our analysis was facilitated by Nielsen and Randall’s framework of factors influencing the implementation of occupational health interventions [73] (see Fig. 1). We chose this model because it attends to psychological and organisational mechanisms that hinder and facilitate desired intervention outcomes. The potential for stigma to impact the adoption and uptake of interventions indicated that the inclusion of psychological mechanisms was potentially important. We operationalised the model using four overarching domains: the intervention activities; implementation strategy; the intervention context; and mental models. Contextual issues relate to the organisational and economic context in which the intervention takes place. Mental models relate to the participants’ readiness for change and their appraisal of the intervention of key stakeholders.

After initial coding, we thematically synthesised [74, 75] data within each domain inductively, which involved reducing the data into relevant themes. These steps were iterative, e.g. in some cases a finalised theme and supporting data fitted best in a different domain of the framework and were moved. This synthesis process was conducted on studies addressing RQ2 and studies set in healthcare settings and SMEs to address RQ3. Only one study was identified for both the construction [76] and ICT [77] sectors, therefore syntheses could not be conducted.

### Study quality assessment

One review author (CP, HS, CL, AO, AD, JCS, FT, BH, LDW, SM) assessed methodological limitations for each study sampled for RQ2 and RQ3, with 15% rated independently by a second reviewer (CP) to ensure consistency. The Mixed Methods Appraisal Tool (MMAT) [78] supplemented by an 8-item process evaluation tool [79, 80] was used for these assessments.

### Assessing our confidence in the review findings

One reviewer (CP, HS, CL, AO, AD, JCS, FT, BH, LDW, SM) used the GRADE-CERQual (Confidence in the Evidence from Reviews of Qualitative research) to assess our confidence in each finding [81]. A second reviewer reviewed assessments and justifications to ensure consistency. Each finding was classified as low, moderate, or high confidence based on the strength of the evidence.

## Results

### RQ1: scope of the research

#### Study selection results

**Fig. 2:**
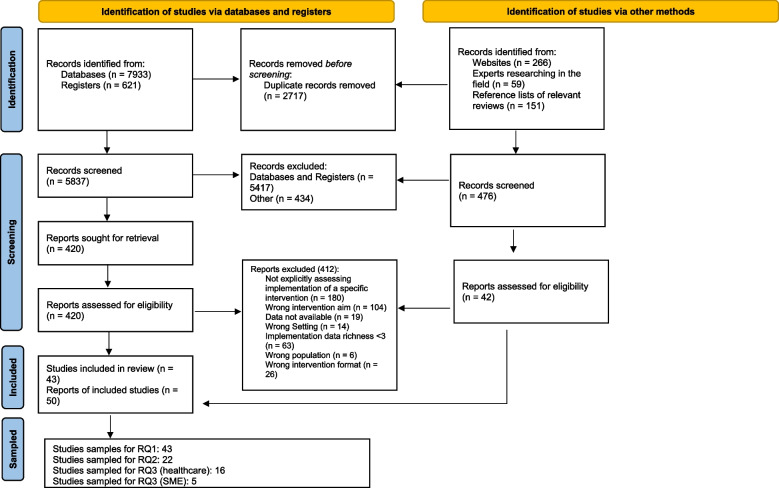
PRISMA 2020 flow diagram. *Adapted from*: Page MJ, McKenzie JE, Bossuyt PM, Boutron I, Hoffmann TC, Mulrow CD, et al. The PRISMA 2020 statement: an updated guideline for reporting systematic reviews. BMJ 2021;372:n71. 10.1136/bmj.n71. For more information, visit: http://www.prisma-statement.org/

We identified a total of 6313 titles and abstracts published between 2009 and 2022. We considered 462 full-text papers. Twelve systematic reviews were identified; however, none of these directly addressed our research questions therefore they were subsequently excluded and instead we used these as a source to identify relevant primary studies. Forty-three primary studies were eligible for inclusion to address RQ1 (see Fig. 2). Reasons for exclusions can be found in Additional file 5.

#### Description of the studies

Studies were conducted mainly in high-income countries. Most studies were conducted in the UK (26%) or Australia (16%) and in large organisations (72%) (Table 3). The studies encompassed a range of private and public sectors most of which were healthcare (40%) or multiple organisations (38%) Few were conducted in construction (*n* = 1) or ICT (*n* = 1). Data extracted from primary studies can be found in Additional file 6.

**Table 3:** Characteristics of included primary studies

Study characteristics (*n* = 43)	Number of studies	Percentage
Study by country		
United Kingdom	11	26%
Australia	7	16%
Canada	4	10%
United States of America	3	7%
Denmark	3	7%
Germany	3	7%
Netherlands	3	7%
Sweden	2	5%
Finland	1	2%
Norway	1	2%
Spain	1	2%
Switzerland	1	2%
Turkey	1	2%
Multiple	1	2%
Unknown	1	2%
Organisations by type		
Healthcare only	17	40%
Multiple organisations	16	38%
Education only	3	7%
Police only	2	5%
Construction only	1	2%
Technology only	1	2%
Prison only	1	2%
Logistics only	1	2%
Utilities	1	2%
Organisation by size		
Large (> 250 employees)	31	72%
Small (< 49 employees) -	3	7%
Mixed	3	7%
Unclear	3	7%
Medium (50–249 employees)	2	5%
Small to large	1	2%

#### Summary of intervention characteristics

All three intervention types, according to the Integrated Workplace Model of Mental Health (protection, promotion, responding), were represented in the included studies with a balanced mix of work-directed and worker-directed interventions (see Table 4). Most of these were interventions that promoted workplace mental health well-being by creating positive aspects of work, such as management behaviours and developing employees’ strengths by building resilience or stress management techniques (*n* = 24). There were fewer studies with an intervention focused on the protection of health by modifying harmful psychosocial working conditions (*n* = 11) and interventions with a focus on responding to mental health problems in individuals when they occurred (*n* = 8). Twenty interventions were mainly work-directed (organisational interventions), 21 were worker-directed (individual interventions) and 2 interventions targeted both, workers and work (integrated interventions).

**Table 4:** Intervention characteristics

Intervention type	Studies
To *protect* mental health by reducing work-related risk factors (*n* = 11) Work-directed intervention (*n* = 11)	• Risk assessments and action planning [82–90] • System engineering for patient safety [91] • Job redesign [92]
To *promote* workplace mental health well-being by creating positive aspects of work, and developing employees’ strengths (*n* = 24) Work-directed intervention *n* = 8 [42, 93–99] Worker-directed intervention *n* = 14 [77, 100–112] Integrated intervention (work- and worker-directed) *n* = 2 [113, 114]	Creating positive work environments • Improving management/leadership competencies, behaviours, or standards [42, 93–99] • Improving team working [115] Developing employees’ strengths • Mindfulness [100–102, 106] • Stress management techniques and resilience enhancement, e.g. achieving life balance, time management, maintaining a positive outlook, relationships, and networking, emotional intelligence styles [77, 103, 107, 108, 112] • Cognitive behaviour therapy to improve coping [105] • Yoga [109] • Recovery promoting activities [110] • Booster breaks [111] Develop health-promoting leadership and work design together with supportive teams, and individual strengths [113, 114]
To *respond* to mental health problems when they occur (*n* = 8) Worker-directed intervention *n* = 8	• Mental health first aid [76, 104, 116–119] • Return to work support: e.g. Cognitive Behaviour Therapy to improve problem-solving [120, 121]

Interventions were mostly delivered face to face (*n* = 28), with a few being delivered online or via a smartphone (*n* = 7) or using multiple methods (*n* = 3) such as face-to-face and online or DVD and telephone. Some studies did not report the intervention delivery mode (*n* = 5).

### RQ2: Qualitative evidence synthesis of barriers and facilitators to implementation

#### Results of the sampling framework

Of the 43 eligible studies identified for RQ1, 22 had data richness of ≥ 4 and were synthesised for RQ2. For RQ3, 16 studies had a data richness ≥ 3 and were set in the healthcare sector, and 5 had a data richness ≥ 3 and were set in SME. Since few were conducted in construction (*n* = 1) or ICT (*n* = 1) syntheses could not be conducted. Overall, there were 35 unique studies included in the analyses. Data extraction can be found in Additional files 7 (RQ2) and 8 (RQ3).

#### Study quality assessment

The quality assigned to the studies’ process evaluation in terms of the reliability of their findings was rated as *high* in 12 studies [76, 82, 92, 100–105, 116, 117, 120], *medium* in 17 studies [42, 93–97, 106–111, 113–115, 118, 119], and *low* in six studies [77, 83, 84, 91, 98, 112].

The quality assigned to studies’ process evaluation in terms of the usefulness of their findings was rated as *high* in 15 studies [76, 82, 92, 97, 100–103, 105, 107, 116–120], *medium* in 16 studies [83, 84, 93–96, 98, 104, 106, 108–114], and *low* in four studies [42, 77, 91, 115]. Further details of the study quality assessment can be found in Additional file 9.

#### Review findings

Multiple factors hindering and/or facilitating implementation were identified and mapped onto Nielsen and Randall’s (73) framework domains. All factors (i.e. barriers and facilitators) were thematically synthesised, creating several themes within each domain, which formed the basis of each finding. An overview of findings and GRADE-CERQual assessment are presented in Table 5. More details, with example quotes, are reported in Additional file 10. CERQual assessments are detailed in Additional files 11, 12, and 13 for each RQ.

**Table 5:** Summary of qualitative review findings and GRADE-CERQual

Review question	Summary of review finding	Contributing studies	CERQual assessment of confidence in the finding	Explanation of CERQUal assessment
Intervention activities				
RQ2: Barriers and facilitators to implementing workplace mental health interventions	Finding RQ2-1: Intervention content related to relevance and tailoring to the needs of the organisation or sector context and the participants.	[82, 83, 93, 94, 97, 98, 103, 107, 113, 116, 117, 119]	High confidence	No major concerns were reported concerning methodology, relevance, coherence, or adequacy. Hence, the evidence collected to support this finding is considered supportive.
	Finding RQ-2. Flexibility and tailoring of intervention delivery related to when, where, and how the intervention was delivered.	[82, 83, 93, 98, 103, 106, 113, 117, 119]	Moderate confidence	Moderate concerns regarding methodological/reporting limitations across most studies and minor concerns regarding relevance due to the overrepresentation of Western countries.
	Finding RQ2-3: Consolidating learning and sustaining knowledge and skills acquired during intervention related to feedback, reminders, refresher sessions, and timing of intervention activities.	[92, 98, 103, 112, 117, 119]	Moderate confidence	Moderate concerns regarding relevance and adequacy, due to very limited data.
	Finding RQ2-4 Fostering Relationships, openness, and confidentiality related to shared language and experiences with credible and relatable instructors, and composition of a participant group.	[82, 92, 94, 97, 100, 108, 112, 116–119]	Moderate confidence	Although there were no minor concerns across 3 components, one component was considered to have moderate concerns, therefore, we have downgraded the overall assessment once
RQ3a: Barriers and facilitators to implementing workplace mental health interventions in the healthcare sector	Finding RQ3a-1: Intervention content related to relevance and tailoring to the needs of the organisation or sector context and the participants.	[91, 92, 104, 110, 117, 120]	Moderate confidence	Studies had minor to moderate methodological concerns with some having no concerns. Good range of countries and interventions and coherence but most studies had limited depth and/or breadth.
	Finding RQ3a-2: Flexibility and tailoring of intervention delivery related to when, where, and how the intervention was delivered.	[84, 91–93, 100, 102, 104, 109, 110, 117, 120]	Moderate confidence	Studies had minor to moderate methodological concerns with some having no concerns. Good range of countries and interventions and coherence but most studies had limited depth and/or breadth.
	Finding RQ3a-3: Accessibility of the intervention related to time required, external ownership of intervention components, availability of intervention providers, and when and where the interventions were delivered	[92, 93, 102, 104, 109, 110, 114, 117, 120]	High confidence	Good number of studies. Good range of countries, organisation size, and range of interventions. Minor or no concerns regarding the implementation methods, with the exception of one moderate. Good coherence. Most studies have good/fair breadth.
	Finding RQ3a-4: Fostering relationships, and openness related to shared language and experiences with credible and relatable instructors and, where relevant, composition of a participant group.	[92, 100, 102, 104, 107, 117, 120]	High confidence	All studies had minor to no concerns regarding methodology. Good coherence. Range of interventions, although limited to seven studies demonstrated good/fair breadth and depth.
	Finding RQ3a-5: Consolidating learning and sustaining knowledge and skills acquired related to feedback, reminders, refresher sessions, and role play.	[102, 109, 117, 120]	Moderate confidence	Limited number of studies although most had minor or no methodological issues. Good coherence and range of interventions across 4 countries. One study lacked depth.
RQ3b: Barriers and facilitators to implementing workplace mental health interventions in SMEs	RQ3b-1: Intervention content related to relevance and tailoring to the needs of the organisation or sector context and participants, varied and easy to use.	[76, 77, 101, 117]	Moderate confidence	There are moderate concerns with one criterion and very minor concerns for the other three criteria, therefore confidence has been marked as moderate
	RQ3b-2 Fostering relationships and openness related to collaboration, communication, and reflective dialogue about mental health.	[76, 117]	Moderate confidence	Marked down due to adequacy judgment despite very minor or minor concerns on the first three categories.
Implementation strategies				
RQ2: Barriers and facilitators to implementing workplace mental health interventions	Finding RQ2-5 Management/leadership buy-in and support at all levels related to prioritisation, proactivity, and promotion of the intervention, appropriate authority, and continuous commitment, demonstrating the value placed on it.	[82, 83, 92–96, 100, 108, 111, 113, 116–119]	High confidence	Large number of studies with no or minor concerns, only one moderate concern. Good range of countries, organisations, and interventions. Most with good depth or breadth.
	Finding RQ2-6: Communication of clear, succinct, relevant, timely, information related to the use of formal, appropriate, and varied channels, to share information both vertically and horizontally at every stage of the implementation.	[82, 83, 92–94, 98, 100, 107, 108, 112, 113, 116, 118, 119]	Moderate confidence	Most studies with no or minor concerns regarding methodology, however, four studies with either serious or moderate concerns. Large number of studies most with good/fair breadth/depth, some with limitations. Wide range of countries, organisations, and interventions.
	Finding RQ2-7. Change agents related to a range of individuals, both internal and external who could drive or champion implementation or the intervention.	[83, 94, 97, 100, 118–120]	High confidence	Fair number of studies most with minor concerns, one with serious concerns regarding methodology and little depth, and another with little breadth/depth. Good range of countries, organisation size, and type.
	Finding RQ2-8. Assistance and backing to engage in the intervention related to support from colleagues, managers, intervention experts, and technology teams for both employee-level participation and for the intervention activities themselves.	[83, 92, 93, 97, 98, 105, 117–119]	High confidence	Fair number of studies, most with minor or no concerns, one with serious concerns regarding methodology and another with little breadth/depth. Good range of countries, organisation size, and type.
	Finding RQ2-9. Stakeholder engagement related to involvement in decisions, reaching consensus, and participator approaches to implementation involving key stakeholders at all levels.	[82, 83, 92, 94, 95, 97, 98, 100, 113, 117, 118]	High confidence	Fair number of studies, most with minor concerns, although three with serious concerns regarding methodology and little depth. Good range of countries, organisation size, and type.
	Finding RQ2-10: Participants’ choice related to voluntary or mandatory participation in the intervention.	[97, 100, 117, 119]	Low confidence	Limited number of studies. There were only minor concerns in all studies regarding methodological limitations, although one study had limited breadth and depth therefore an overall score of minor confidence has been allocated.
	Finding RQ2-11: Clarity of roles, responsibilities, and boundaries relate to local implementation and intervention providers.	[93, 103, 107, 119]	Moderate confidence	There were none to minor concerns in all studies regarding methodological limitations. However, a limited number of studies and only two studies had good/fair breadth and depth, therefore a score of moderate confidence has been allocated
	Finding RQ2-12: Coherence with the organisations values, policies, and structures related to ability to integrate or embed the intervention into the organisation. The capacity of an intervention to align and integrate responsively to an organisation’s existing internal policies and alongside on-going initiatives is imperative.	[82, 83, 92, 94, 98, 106, 108, 111, 113, 116, 118]	Moderate confidence	There were concerns in most studies regarding methodological limitations, including one with serious and one with moderate concerns. Mixed sectors. Most studies had good/fair breadth and depth therefore overall score of moderate confidence has been allocated.
	Finding RQ2-13: Intervention initiation related to impact and strategy for wellbeing.	[82, 116, 119]	Moderate confidence	This finding only included three studies however, the finding was very descriptive, one study included a range of organisation types and sizes and there were minimal methodological concerns, therefore the overall score of moderate confidence was given
RQ3a: Barriers and facilitators to implementing workplace mental health interventions in the healthcare sector	Finding RQ3a-6: Management/leadership buy-in and support at all levels related to prioritisation, proactivity, and promotion of the intervention, appropriate authority and continuous commitment, appropriate authority, and demonstrating the value placed on it.	[84, 92, 93, 96, 100, 105, 110, 117, 120]	High confidence	Most studies had no or minor concerns with methodological limitations. Good range of countries and interventions. Good coherence and most studies fair to good breadth and depth.
	Finding RQ3a-7. Communication of clear, succinct, relevant, timely information related to the use of formal, appropriate, and varied channels, to share information both vertically and horizontally at every stage of the implementation	[84, 91–93, 100, 107, 110, 117, 120]	High confidence	Most studies have no or minor methodological limitations, although two with moderate concerns. Good mix of countries, and interventions, mostly large healthcare organisations. Good coherence. Four studies with some limitations regarding depth.
	Finding RQ3a-8: Change agents related to a range of individuals, both internal and external who could drive or championed implementation.	[84, 100, 110, 120]	Moderate confidence	Some methodological limitations and a limited number of studies with some breadth and depth issues.
	Finding RQ3a-9: Assistance and backing to engage in the intervention related to support from colleagues, managers, intervention experts, and technology teams for both employee-level participation and for the intervention activities themselves.	[84, 92, 93, 105, 107, 109, 110, 117, 120]	Moderate confidence	Most studies had few methodological limitations and a fair range of interventions and countries. Moderate concerns for a limited number of studies and breadth or depth issues.
	Finding RQ3a-10: Stakeholder engagement related to collaborative working, involvement in decisions, and participator approaches to implementation involving key stakeholders.	[84, 91, 92, 110, 120]	Moderate confidence	Some methodological limitations and a limited number of studies with some breadth and depth issues.
	FindingRQ3a-11: Clarity of roles, responsibilities, and boundaries relate to local implementation and intervention providers.	[93, 107, 120]	Moderate confidence	Limited number of studies with 2/3 UK-based, range of diverse interventions, although all delivered face to face. Few methodological limitations.
RQ3b: Barriers and facilitators to implementing workplace mental health interventions in SMEs	RQ3b-3: Management/leadership buy-in and support related to promoting and engaging employees in intervention.	[77, 101, 117]	Moderate confidence	Moderate confidence due to adequacy judgment and two other minor concerns.
	RQ3b-4: Promoting participation in the intervention related to multiple promotion strategies to increase local visibility through change agents and managers.	[76, 77]	Moderate confidence	It is likely that this review finding is a reasonable representation of the phenomenon of interest. It is hampered somewhat by a lack of depth/richness of data to substantiate, however, as the findings are descriptive in nature, we have given this moderate confidence (despite two components being marked as having moderate concerns).
	RQ3b-5: Clarity of roles, responsibilities, and boundaries relate to local implementation and intervention providers.	[76, 77]	Moderate confidence	It is likely that this review finding is a reasonable representation of the phenomenon of interest. It's hampered somewhat by a lack of depth/richness of data to substantiate it and some methodological limitations, however, it is fairly descriptive in nature.
Context				
RQ2: Barriers and facilitators to implementing workplace mental health interventions	Finding RQ2-14: Workload demands related to excessive and poorly managed workloads negatively impact implementation.	[83, 92, 93, 96–98, 100, 103, 106, 111, 116, 119]	Moderate confidence	Good number of studies with good depth and breadth, highly reliable and useful. The range of counties was limited and two studies with serious methodological limitations.
	*Finding RQ2-15a: Internal resources: staffing* related to staffing across all levels of an organisation which had the potential to influence implementation of interventions.	[83, 92, 95–97, 113]	High confidence	Moderate number of studies with a good range of countries, interventions and organisations, Few methodological limitations (only one serious), and most with good depth and breadth.
	*Finding RQ2-15b: Internal Resources: Time* related to organisations affording participants in intervention activities with the time to participate in the activities, and flexibility within pre-existing standardised scheduling practices.	[83, 94, 97, 98, 105–108, 111, 116, 118]	High confidence	Large number of studies (*n* = 11) across a range of countries, organisations, and interventions. Only two studies had methodological limitations and therefore low reliability.
	*Finding RQ2-15c: Internal resources: physical environment* related to the availability and willingness of the organisations to dedicate adequate and appropriate space to meet the needs of the intervention.	[100, 103, 105, 107, 116]	High confidence	Moderate number of studies with good range of countries, interventions and organisations. Non or minor methodological limitations and all with good depth and breadth.
	*Finding RQ2-15d: Internal resources: financial* related to organisations possessing the necessary financial resources to support the intervention.	[83, 97, 100]	Low confidence	Few studies one with serious methodological limitations, a small range of countries and organisations.
	Finding RQ2-16: Organisational Change/Stability related to significant change or transitions within an organisation.	[82, 94, 95, 97]	Moderate confidence	Few studies although all with only minor methodological limitations. Range of countries and organisations limited.
	Finding RQ2-17: Culture alignment related to the alignment of the culture of the organisation relative to the aims and objectives of the intervention activities.	[83, 97, 98, 108, 111]	Moderate confidence	Limited number of studies. There were serious methodological concerns in three and two had limited depth.
RQ3a: Barriers and facilitators to implementing workplace mental health interventions in the healthcare sector	FindingRQ3a-12: Internal resources**:** time-related to time to plan, integrate, and engage in the implementation and delivery of the intervention	[84, 91–93, 96, 100, 105, 107, 109, 110, 114, 120]	High confidence	Most studies have no or minor concerns. Good range of interventions and countries, and large healthcare organisations. About half the studies have good/fair breadth and depth.
	Finding RQ3a-13: Internal resources: physical environment related to appropriate and accessible intervention location.	[100, 104, 105, 107, 110, 114]	Moderate confidence	No or minor methodological issues. Although limited number of studies, a good range of interventions is good and fair breadth and depth of most studies.
RQ3b: Barriers and facilitators to implementing workplace mental health interventions in SMEs	RQ3b-6: Physical environment related to appropriate and accessible intervention location.	[101]	Low confidence	Our confidence in this finding is minor because we have moderate concerns across three of four components, and one component judged to have serious concerns.
Mental models				
RQ2: Barriers and facilitators to implementing workplace mental health interventions	Finding RQ2-18a; Experience and awareness of mental health issues related to level of engagement with a workplace mental health intervention.	[94, 103, 105, 106, 108, 117–119]	High confidence	Only minor methodological concerns and minor concerns regarding coherence due to some interpretation in the finding, but overall, the evidence is clear and supportive, and the finding is largely descriptive.
	Finding RQ2-18b. Experience with previous organisational initiatives. related to expectations and attitudes towards the intervention.	[83, 100, 105, 112]	Moderate confidence	We have concerns about the methodological limitations of some of the contributing studies and concerns about the thickness of the data across all studies, therefore, we have downgraded once.
	Finding RQ2-19: Perception of intervention as motivation to engage related to participants’ relatability and level of interest in workplace stress, the positive reputation of the intervention perceptions of the usefulness and progress made.	[82, 92, 96, 98, 100, 106, 111, 116, 117, 119]	Moderate confidence	Overall, there were moderate concerns relating to methodological limitations, minor concerns regarding relevance, and no-to-minor concerns regarding coherence and adequacy. A decision was made to grade these findings as offering moderate evidence, in relation to significant methodological limitations, that either indicated bias or did not adequately describe the methodology, thereby limiting our ability to assess the reliability of the findings. Moreover, the study findings were based on high-income countries, and large organisations, limiting generalisability.
	Finding RQ2-20: Stigma about mental health and perceived confidentiality issues related to participants willingness to engage in the intervention, be open, and talk about their experiences or gain insight into mental health problems.	[97, 105, 107, 116–119]	High confidence	The confidence levels for methodological limitations were minor, while relevance, coherence, adequacy were all related as no/minor concerns, warranting an overall assessment grade of high confidence.
RQ3a: Barriers and facilitators to implementing workplace mental health interventions in the healthcare sector	Finding RQ3a-14: Previous experience of mental health interventions and training related to a positive or negative experience of intervention or training	[100, 105]	Moderate confidence	
	Finding RQ3a-15: Perception of intervention as motivation to engage related to participants’ relatability and level of interest in workplace stress, perceptions of the usefulness and positive reputation, of the intervention, and progress made.	[84, 91–93, 100, 102, 105, 109, 110, 117, 120]	High confidence	Few methodological limitations, a good range of countries, interventions, and large healthcare organisations, good coherence. Some breadth and depth issues for some studies
	Finding RQ3a-16: Stigma about mental health and perceived confidentiality issues related to participants’ willingness to engage with the intervention, be open, and talk about their experiences or gain insight into mental health problems.	[84, 102, 104, 105, 107, 114, 117]	High confidence	Few methodological limitations. Good range of countries and interventions, good coherence, and most studies have good/fair breadth and depth.
RQ3b: Barriers and facilitators to implementing workplace mental health interventions in SMEs	RQ3b-7 Perception of intervention as motivation to engage related to participants’ relatability and level of interest in workplace stress, positive reputation of the intervention, perceptions of the usefulness, and progress made	[42, 76, 77, 101, 117]	Moderate confidence	We have some concerns about some components, therefore, we have marked our confidence as moderate.

### Intervention activities

Synthesised findings within the ‘intervention activities’ domain included: intervention content; flexibility and tailoring of intervention delivery; consolidating learning and sustaining knowledge; fostering good relationships, and a culture of openness.

Nine studies [82, 83, 93, 98, 103, 106, 113, 117, 119] reported factors on flexible intervention delivery in RQ2. A range of delivery modes [83, 98, 103, 106, 119], and lengths [93, 113] of delivery were preferred by different participants. Flexibility was perceived positively [93, 113, 119].

Six studies reported factors associated with consolidating and sustaining knowledge [92, 98, 103, 112, 117, 119]. Barriers included time between intervention sessions and ownership of the intervention (or components of the intervention) by external companies or consultants which can restrict or prevent access to intervention materials in the longer term [92, 112], whilst facilitators included reminders and refresher sessions [98, 103, 117, 119].

The intervention content relevance and tailoring were identified in 11 studies [83, 93, 94, 97, 98, 103, 107, 113, 116, 117, 119]. This included tailoring intervention contents for the organisation, sector, or individual as facilitative [83, 93, 97, 113, 116, 117, 119], while not considering the relevance of the intervention to the contextual issues was hindering [107, 116].

Fostering relationships and a culture of openness was mentioned by 11 studies [92, 94, 97, 100, 108, 112, 116–119]. Facilitating factors included, attending interventions with colleagues [98, 100, 108, 112], credible and relatable instructors [97, 108, 116, 118, 119] sometimes with lived experience of mental health issues or the sector [108, 119], and using a shared language [108, 112]. Barriers included instructors lacking compassion [119], and unclear language [92]. Group interventions including mixed levels of seniority [108, 117] and having internal or external intervention providers [94, 97, 116] were seen as both a barrier and facilitator to developing a culture of openness.

### Implementation strategy

The themes identified within the implementation strategy included: management/leadership buy-in and support; communication of clear and succinct information; change agents; assistance and backing to engage in the intervention (practical support); stakeholder engagement; participant choice (voluntary versus mandatory); clarification of roles, responsibilities, and boundaries; coherence with the organisations values, policies, and structures; and intervention initiation.

Management/leadership buy-in and support were reported in 14 studies [83, 92–96, 100, 108, 111, 113, 116–119]. Leadership support from every level [83, 94, 100] to endorse, prioritise, and promote the value of the intervention [83, 93, 95, 100, 103, 108, 113, 116, 117, 119] facilitated implementation, while a lack of leadership or managerial support hindered implementation [96, 97, 108, 116, 118, 119].

Communication (*n* = 13) [83, 92–94, 98, 100, 107, 108, 112, 113, 116, 118, 119] about the value, need, benefit, and accessibility of the intervention was facilitative [83, 100, 108, 119], while a lack of communication was a barrier [93, 112, 116, 118]. Using existing [100] and varied [92, 94, 107, 118, 119] communication channels was beneficial as was regular communication [83, 94, 98, 107, 113].

Various change agents were identified in several studies (*n* = 8) [83, 94, 97, 100, 118–120] as key facilitators of implementation. Further, different forms of support were reported across studies [97, 105, 113, 117, 119], e.g. sharing information or lacking [92, 93, 98, 105, 107, 118, 119], e.g. minimal technical support.

Stakeholder engagement was reported by 10 studies [83, 92, 94, 95, 97, 98, 100, 113, 117, 118] as facilitating implementation, for example, via multidisciplinary stakeholder groups [94, 97, 98, 113] and engaging staff at all levels in decision making [83, 92, 94, 95, 103, 113, 118].

Participant choice was discussed in four studies [97, 100, 117, 119]. Voluntary participation was valued [100]; however, for some sectors, mandatory participation may be beneficial [97, 119].

Four studies reported on roles and responsibilities [93, 103, 107, 119], clearly established roles and responsibilities helped implementation [107], while a lack of clarity was a barrier [93, 103, 119].

Intervention integration with the organisation was highlighted by six studies [94, 98, 108, 111, 116, 118]. Overall, interventions contradicting usual organisational values/policies and structures can cause barriers [111, 116], while those that align can facilitate implementation [108, 118]. Finally, two studies reported reasons for intervention initiation, including recognition of the impact of mental health issues [116], and alignment with organisational strategy [119].

### Context

Themes identified in the context domain included: workload demands; availability of internal resources; organisational stability; and cultural alignment. Twelve studies mentioned the role of workload demands, in particular those that were excessive or poorly managed as having a negative impact on implementation [83, 92, 93, 96–98, 100, 103, 106, 111, 116, 119].

Four subthemes emerged pertaining to the availability of internal resources to support implementation: staffing levels [83, 92, 95–97, 113], the affordability and flexibility of time provision [83, 94, 97, 98, 105–108, 108, 111, 116, 118], the adequacy and availability of suitable physical environments for intervention activities [97, 100, 103, 105–107, 116], and financial resources to support the intervention [83, 97, 100]. Four studies outlined the influence of organisational stability or change on implementation activities [82, 94, 95, 97]. Finally, the extent to which the culture of the organisation relative to the aims and objectives of the intervention activities was found to influence implementation across five studies [83, 97, 98, 108, 111].

### Mental models

Themes identified in the mental model’s included: previous experience and awareness of mental health issues; previous experience of organisational initiatives; perception of intervention (usefulness) as motivation to engage; and stigma about mental health issues perceived confidentiality issues.

Previous experience or awareness of mental health issues can facilitate engagement [108, 118, 119], while a lack of awareness is hindering [94, 105, 106]. Additionally, previous negative experiences of organisational initiatives can negatively impact expectations [83, 105, 112], while previous positive experiences of similar interventions facilitate positive attitudes [100].

Various factors affecting motivation to use the intervention were reported in nine studies [92, 96, 98, 100, 106, 111, 116, 117, 119]. Curiosity, perceived usefulness, and progress towards resolution were facilitators [92, 100, 106, 117], while a lack of self-discipline, interest, and perceived usefulness of the intervention were barriers [96, 98, 111, 116].

Finally, seven studies reported stigma [97, 105, 107, 116–119], as a barrier to engagement [97, 105, 107, 116]. Sharing personal stories and open dialogue about mental health was a facilitator to overcoming stigma and engagement [117, 119].

For RQ2, facilitators graded as having ‘high confidence’ included relevant and tailored programme content, continuous and pro-active leadership buy-in and support, internal or external change agents and champions as drivers of change, assistance, and backing-up by managers and peers, resources, and experience and awareness with mental health issues.

For RQ3a, in the health care sector, specific facilitators were identified as easy accessibility of intervention with time provided, fostering relationships with instructors, and where relevant, peers, clear communication, and perceptions of intervention. Stigma and confidentiality issues were reported as barriers overall (high confidence). Due to a lack of studies in construction and ICT, separate analyses were not possible for these sectors. For RQ3b, SMEs, the only additional finding (of moderate confidence) was within the domain of implementation strategies and reported on ‘promoting participation in the intervention’.

The findings answering research questions two and three were mostly thematically consistent across all syntheses. Only a few findings from RQ2 do not apply to our findings within the healthcare sector (RQ3a) or SMEs (RQ3b), i.e. voluntary participation and intervention initiation.

### Review author reflexivity

In keeping with quality standards for reflexivity within qualitative research, we maintained a reflexive stance throughout all stages of the review process. We consider how our views and beliefs could influence the choices we make in relation to the scope of the review and our review methods, our interpretation of the data, and our findings. The review team is from varied professional backgrounds, many with mental health expertise and experience in qualitative research and systematic reviews. Core review authors (BA, BG, MM, and CL) have experience in implementation science, with (CP, HS) from nursing and psychology backgrounds with experience undertaking systematic reviews. Other authors (AO, FT, JCS, GP, LW, NF, AD, BH, PC, SM, UH, EA) provided a quality review role. During each stage of the entire review, the team constantly referred to each other to resolve conflict, making team decisions that reflected the multi-disciplinarity of the team members with backgrounds in diverse theories and methods. Using this approach, we believe that our classification of barriers and facilitators and the interpretation of the different studies are comprehensive and reduce the influence of individual researcher subjectivity.

## Discussion

This review set out to establish the scope of intervention studies investigating the implementation of workplace mental health promotion interventions (RQ1) and to synthesise the evidence of barriers and facilitators to implementation (RQ2) with a specific focus on the construction, healthcare, and ICT sectors (RQ3a) and on SMEs (RQ 3b) using studies that report rich data.

We discovered limited implementation evidence specific to the construction and ICT sectors. We also found that whilst a range of different workplace mental health interventions exists, most interventions targeted individual workers with a wide scope of programme types, for example, mindfulness training, yoga classes, and mental health first-aid opportunities. A smaller number of studies targeted the improvement of psychosocial working conditions or the work environment as part of an organisational intervention, including action planning based on risk assessments or supervisor capacity training.

Factors affecting implementation were identified across all Nielsen and Randall framework [73] domains. Our findings showed several barriers and facilitators that were judged to be well supported by the studies. In relation to RQ2, high-confidence findings pointed to aspects of the intervention itself such as relevance and tailoring, and mental models that related to experience and awareness of mental health issues appeared to be a facilitator, whereas stigma and confidentiality issues appeared as barriers.

One of the key findings of our review was the importance of supervisors and senior management in the implementation process. Although classified under the domain of ‘implementation strategies’, manager support can affect different domains represented in the Nielsen and Randall model [73] and influence the specific intervention activities, context, and mental models. Our findings are in line with research, that has repeatedly stressed the importance of line managers and senior management to support or obstruct workplace mental health interventions [122–124]. Managers usually have a great responsibility in implementing mental health interventions and are key players in allocating resources for interventions and continued support for sustained delivery, encouraging the uptake of such programmes [26, 125]. Lack of supervisor and manager support and adoption of interventions can be reflective of a range of reasons, such as lack of awareness and knowledge about mental health; limited skills for how to approach individuals with symptoms of compromised mental health [126]; limited competencies to modify psychosocial working conditions; managers’ own stressful working conditions and limited decision latitude [127] and competing interests for the use of staffing and financial resources [26]. Scholars therefore stressed the importance of manager and supervisor training as part of the intervention and implementation process [125, 128, 129] alongside building specific implementation ‘capacity’ [130].

The importance of mental models was another clear result of our review, particularly awareness of mental health issues, mental health stigma, and participants’ concerns about confidentiality. Stigma and confidentiality issues were highlighted as barriers for help-seeking and engaging in the intervention [71, 82]. Interestingly, only a few of the studies included an explicit anti-stigma component as part of their intervention. Research evidence although methodologically limited, highlights that workplace anti-stigma interventions can positively influence knowledge, attitudes, and supportive behaviour towards people with mental illness [131]. Anti-stigma components may form an important aspect of the implementation strategy of workplace mental health interventions [132].

To our knowledge, this is the first review to distil specific barriers and facilitators for implementation specific to sectors (we included healthcare, construction, and ICT) and specifically for SMEs. While some studies used samples from multiple organisations, including construction and ICT among other sector organisations, the results were not specifically reported by sector or organisation, making a meaningful synthesis not possible.

Nevertheless, we identified enough studies to assess barriers and facilitators in health care and distil results rated at a ‘high confidence’ level. Many findings identified for research question 2 were also found in studies conducted in health care; however, certain findings seem to be particularly important for implementation in this sector, e.g. accessibility of the intervention, clear communication, timely and relevant information, and time to plan and engage in the implementation all pointing to the fact that time pressure is a specific challenge. In addition, since most employees work in shift systems, clear communication and accessibility of information is crucial. Also here stigma about mental health was identified as a barrier, confirming research showing that mental health stigma is also widespread among healthcare workers [133], even though many nurses and physicians suffer from mental health problems like burnout themselves [134, 135].

In relation to specific barriers and facilitators for the implementation in SMEs, the low number of studies limited the level of confidence in findings to ‘moderate’ or ‘low’. While all identified barriers and facilitators were similar to the general findings, one particular factor deserves further discussion, namely leadership support for the intervention. It has been noted that SME managers and owners experience particular challenges in implementation due to the variety of professional roles and responsibilities with high levels of stress [136]. As an important detail of our findings, particularly in SMEs, the lived experience of supervisors experiencing workplace mental health issues was a facilitator to engaging in the intervention and driving change [42, 137]. The study by Moll et al. [117] highlighted this as a significant facilitator for employee participation, when leaders talked openly about their own mental health problems, thereby creating an open and non-judgemental dialogue. Scholars have argued that particularly in SMEs, leaders can serve as a ‘contagion’ of good mental health due to their proximity to employees [137].

This review complements the findings of other reviews about barriers and facilitators of workplace health interventions [26, 30]. Compared to the review by Yarker et al. [26] that was restricted to studies published between 2019 and 2021, our review covers a larger time span (2009–2022) and also identified more but mostly different studies (only 5 studies identified in Yarker et al. were also identified in our review) as we included all study designs, while Yarker et al. only included qualitative studies. The review by Daniels et al. [30] identified studies published between 2009 and 2018, thereby not including studies up to 2022, which are included in our review. However, using a much broader approach in their search including all studies that report on the implementation and effects on psychological well-being, regardless of the intended focus of the intervention, they identified a much larger number of studies (74 studies described in 117 papers). Nevertheless, again the overlap with the studies identified in this review is small (around 30% of the studies identified in our review are included in the reviews by Daniels et al.). All three reviews use a different framework for synthesizing the results, thereby focussing on somewhat different aspects. For example, while Daniels et al. developed their own coding frame based on prior systematic reviews and frameworks, Yarker et al. used the Implementation Outcome Framework by Peters et al. [138] and a qualitative meta-synthesis approach. Despite all these differences, the three reviews show similarities in their findings including the importance of continuous and pro-active leadership buy-in and support, as well as the need for relevant and tailored content of the intervention. In addition, each review further investigates different aspects according to their specific approach. Daniels et al. also highlight the essential role of mechanisms (often associated with social factors) that need to be activated for the intervention to be implemented, while Yarker et al. point to the essential role of appropriate facilitator training. However, none of the other reviews investigated implementation barriers and facilitators specifically for SMEs and within specific sectors such as the healthcare sector.

Our review has relevance for both practice and research into addressing workplace mental health. The review was conducted during the formation of the MENTUPP intervention and was used to shape the development of the intervention and its implementation. The findings can also be relevant to other workplace mental health interventions and their implementation. The results of this review therefore should be used to improve the implementation of mental health interventions at work. However, this review also identified important knowledge gaps about implementation in SMEs and male-dominated sectors.

### Strengths and limitations

This review follows good practice in conducting and reporting systematic scoping reviews [45–47] and meta-synthesis [48, 49, 139]. A particular strength is the comprehensive and rigorous search strategy using 11 databases with the inclusion of the grey literature. Consultation with experts and stakeholders, in accordance with good practice for conducting systematic reviews [140, 141], allowed us to capture any additional studies within the scope of our search strategy. Another strength was the transdisciplinary approach to evidence synthesis guided by established frameworks used in implementation science [73, 142]. Further, the evidence synthesis focussed on findings presented in the results sections of the original articles and did not use information or statements provided by the authors in discussion or conclusion sections to ensure that the synthesis was not biased by values or subjective views of the original authors.

Some limitations of the review need to be considered. Our searches were limited by date to reflect implementation issues in modern workplaces. However, we may have excluded important studies published prior to 2009. The assessment tool for the quality of included studies was not designed to assess researcher reflexivity, which is key to understanding the results of qualitative studies [143]. We were therefore not able to gauge the level of influence the researchers may have had on the individual study process and outcome. We used unevaluated filter terms to search the literature, which is considered experimental [144]. However, other recommended methods for searching for implementation evidence, e.g. shifting the identification of included studies from the search process to the sifting process [144], were not feasible given the breadth of interventions that were included in this review, combined with available review resources. To strengthen our identification process, we applied additional approaches. For example, we (i) contacted authors of studies included in related effectiveness reviews to identify potential process evaluations, (ii) we contacted active researchers in the field and asked them to review our list of included studies and suggest other possible studies, and (iii) we reviewed reference lists of relevant reviews.

### Current and future work

The limited number of organisational-level interventions with process evaluation identified in this review is in line with findings reported in other overviews [26, 30] and has been critically commented on by several scholars [9, 145] asking for more organisational-level interventions with a thorough evaluation. Further research may also focus on the question of intervention-type-specific barriers and facilitators. It can be hypothesised, that obstacles to implementation differ between organisational and individual interventions [146]. Whereas stigma, confidentiality, and disclosure issues may play a predominant role in individual interventions, it can be expected that issues related to organisational power struggles and definitions of roles may be relevant for organisational interventions. However, this review did not include enough interventions and sufficient data richness to fully examine implementation factors associated with intervention focus (protection, promotion, treatment) or delivery mode. More detailed knowledge that can help to address the specific barriers and facilitators for specific interventions and delivery modes would be beneficial.

## Conclusion

The results of this review provide high-confidence evidence of barriers and facilitators to implementation of mental health interventions at work which could serve as guidance when designing intervention studies. Nevertheless, the review also shows that we know most about implementation in large organisations in high-income countries and in the healthcare sector. There is an absence of implementation evidence in the ICT and construction sector and a dearth of evidence in SMEs which should be addressed in future research.

### Supplementary Information

Additional file 1. PRISMA-ScR and ENTREQ checklists.

Additional file 2. Differences between the protocol and review

Additional file 3. Search strategy.

Additional file 4. Bespoke data extraction template.

Additional file 5. Studies excluded at full review with reason.

Additional file 6. Data Extraction Research Question 1.

Additional file 7. Data Extraction Research Question 2

Additional file 8. Data Extraction Research Question 3.

Additional file 9. Methods used to assess confidence in the review.

Additional file 10. Themes for barriers and facilitators to implementing mental health intervention in the workplace.

Additional file 11. RQ2 GRADE-CERQual Assessments.

Additional file 12. RQ3a (Healthcare) GRADE-CERQual Assessments.

Additional file 13. RQ3b (SME) GRADE-CERQual Assessments.

## Data Availability

The datasets used and/or analysed during the current study are available either in the additional files or available from the corresponding author on reasonable request.

## Abbreviations

CERQual: Confidence in the Evidence from Reviews of Qualitative research
ENTREQ: The Enhancing transparency in reporting the synthesis of qualitative research
EPOC: Effective Practice and Organisation of Care
IGLOO: Individual-group-leaders-organisation-outer context
MENTUPP project: Mental Health Promotion and Intervention in Occupational Settings
MMAT: Mixed Methods Appraisal Tool
MRC: Medical Research Council
PRESS: Peer Review of Electronic Search Strategies
PRISMA: Preferred Reporting Items for Systematic Reviews and Meta-Analyses
QES: Qualitative evidence synthesis
RE-AIM: Reach, Effectiveness, Adoption, Implementation and Maintenance
RQ: Research questions
SMEs: Small-to-medium-sized enterprises
TIDieR: Template for intervention description and replication
WHO: World Health Organisation
