# Supplementary material for: Barriers and facilitators to implementing workplace interventions to promote mental health: qualitative evidence synthesis
Source: Syst Rev. 2024 Jun 7;13:152. doi: 10.1186/s13643-024-02569-2 (PMC11157821; doi:10.1186/s13643-024-02569-2)
Supplement: Supplementary file 2 — Additional file 2. Differences between the protocol and review [file 13643_2024_2569_MOESM2_ESM.docx]

**Additional File 2: Differences between the protocol and review**

**Study Design**

In our protocol, we stated that out review was a systematic scoping review, following Arksey & Malley (2) and Levac’s (3) framework as we were aiming to map the evidence base assessing the implementation of workplace mental health promotion interventions. After identifying the relevant evidence base, we recognised that guidelines for qualitative evidence synthesis (QES) (4–6) were better suited to answer research questions 2 and 3 (RQ2 and RQ3), as we planned to synthesise text that had been extracted verbatim from studies. As such, we have now described this work as a scoping review, relating to RQ1, and a QES, relating to RQ2 and RQ3.

**Criteria for considering studies for this review**

**Study designs**

We said we would include all study design that explicitly investigated, reported or discussed, in the title or abstract, any aspect of implementation of mental health promotion interventions delivered in the workplace. Instead, we excluded studies that just discussed implementation without a pre-planned assessment of intervention implementation).

**Setting**

In our protocol, we said that we would categorise geographical locations based on relevance to Europe and Australia during data charting. Instead, we coded data by country, but did not categorise these countries in relation to Europe and Australia.

**Outcomes of interest**

We said we were going to categorise implementation outcomes during the data charting process. Instead, we extracted implementation outcomes measures used, but we did not chart them.

**Information sources**

In our protocol, we reported that we would search the Institute of Occupational Safety and Health (IOSH) in step two. However, as this is a source for grey literature, we have reported this source in step three in the review.

Our protocol reported that, in step three of our stepwise approach, we would search Grey Matters and reference lists of published guidelines and included studies. However, due to time constraints and the volume of relevant studies identified from other sources, we did not complete these searches.

**Study selection**

In the protocol, we stated that titles would initially be screened, followed by abstracts, however, this process was done together.

**Sampling of studies**

After identifying studies to include in the review, we realised that we had too many studies to include in the qualitative evidence syntheses for aim 2. As such, we followed guidelines to sample a selection of studies for RQ2 and RQ3. Details of the study sampling methods are reported in the main manuscript.

**Charting the data, Data extraction, Data coding**

In the protocol, we said that CP and CL would extract data, however, additional reviewers joined the team during the review, therefore additional reviewers extracted and coded data (HS, AO, AD, JCS, FT, BH, LDW, SM).

In the protocol, we stated that we would structure implementation data (e.g., direct quotes, page numbers) using and adapted version of the RE-AIM framework (7), which has been complemented using selected categories from Nielson and Randall’s model of organisational-level interventions (8) and Moore’s sustainability criteria (9). However, while testing the data extraction sheets, it became clear that the RE-AIM framework is best used to describe domains relating to implementation outcomes (10). This was also true of Moore’s sustainability criteria (9). Nielson and Randall’s (8) framework, on the other hand, describes domains relating to modifiable factors that explain and can enhance implementation. As such, in order to identify and organise barriers and facilitators to intervention implementation (RQ2 and 3), as opposed to implementation outcomes, the review team decided to code extracted quotes using Nielson and Randall’s framework (8).

**Quality assessment of included studies**

In the protocol we said we would use the Mixed Methods Appraisal Tool (MMAT) (11). Since registering and publishing the protocol, we decided to supplement the assessment of methodological quality with the 8-item process evaluation tool (12,13).

Further, in the protocol we stated that ‘Methodological quality will be rated by two reviewers (CL and CP). Additional reviewers joined the review team (AO, AD, JCS, FT, BH, LDW, SM), who contributed to the quality assessment, as well as the data extraction. Therefore, all studies were assessed by one reviewer, with 15% independently rated by a second reviewer. Disagreements were discussed and resolved. A third reviewer was consulted where necessary.

**Assessing our confidence in the review findings**

In our protocol, we did not state how we would assess our confidence in our findings. Following guidance Lewin et al. (14), we used the GRADE-CERQual (Confidence in the Evidence from Reviews of Qualitative research) approach. See the main manuscript for more detail.

**Collating, summarising and reporting**

In the protocol, we said we would code the stage of the intervention using the MRC framework, however, we decided that this did not add value to the review. We also stated that ‘to answer questions 2 and 3, barriers and facilitators will be categorised according to the RE-AIM framework (7), modified using Nielson & Randall’s (8) model for evaluating organisational-level interventions and Moore’s sustainability criteria (9). As described above, to identify factors influencing implementation, we decided that Nielson and Randall’s framework was best suited during the extraction process. Reporting of our findings were therefore structured in line with Nielson and Randall’s overarching domains using summary of finding tables supported by narrative.

After initial coding, we used thematic synthesis (15,16) to look for patterns in the data and bring themes together. This was not reported this in the protocol.

The protocol also stated that ‘We will present tabulated data by sector and then occupational level (i.e. organisational, managerial, etc.) and intervention type’. Instead, we have considered these factors in our findings.

The protocol also says that ‘If the evidence allows, to further answer research question three, we will present tabulated data from included studies focusing specifically on SMEs using the same format’. Unfortunately, there were too few studies set in SMEs, therefore we collated findings from studies set in SMEs without sector specificity, and separately collated findings from studies set in the healthcare. There were too few eligible studies set in the ICT and construction sectors to allow for sector specific syntheses.

References

1. Pollock A, Campbell P, Cheyne J, Cowie J, Davis B, McCallum J, et al. Interventions to support the resilience and mental health of frontline health and social care professionals during and after a disease outbreak, epidemic or pandemic: a mixed methods systematic review (Review). COCHRANE DATABASE Syst Rev. 2020;(11).

2. Arksey H, O’Malley L. Scoping studies: towards a methodological framework. Int J Soc Res Methodol. 2005 Feb 1;8(1):19–32.

3. Levac D, Colquhoun H, O’Brien KK. Scoping studies: advancing the methodology. Implement Sci. 2010 Sep 20;5(1):69.

4. Cargo M, Harris J, Pantoja T, Booth A, Harden A, Hannes K, et al. Cochrane Qualitative and Implementation Methods Group guidance series-paper 4: methods for assessing evidence on intervention implementation. J Clin Epidemiol. 2018 May;97:59–69.

5. Harris JL, Booth A, Cargo M, Hannes K, Harden A, Flemming K, et al. Cochrane Qualitative and Implementation Methods Group guidance series—paper 2: methods for question formulation, searching, and protocol development for qualitative evidence synthesis. J Clin Epidemiol. 2018 May 1;97:39–48.

6. Flemming K, Noyes J. Qualitative Evidence Synthesis: Where Are We at? Int J Qual Methods. 2021 Jan 1;20:1609406921993276.

7. Glasgow RE, Harden SM, Gaglio B, Rabin B, Smith ML, Porter GC, et al. RE-AIM Planning and Evaluation Framework: Adapting to New Science and Practice With a 20-Year Review. Front Public Health [Internet]. 2019 [cited 2022 Dec 12];7. Available from: https://www.frontiersin.org/articles/10.3389/fpubh.2019.00064

8. Nielsen K, Randall R. Opening the black box: Presenting a model for evaluating organizational-level interventions. Eur J Work Organ Psychol. 2013;22(5):601–17.

9. Moore JE, Mascarenhas A, Bain J, Straus SE. Developing a comprehensive definition of sustainability. Implement Sci. 2017 Sep 2;12(1):110.

10. King DK, Shoup JA, Raebel MA, Anderson CB, Wagner NM, Ritzwoller DP, et al. Planning for Implementation Success Using RE-AIM and CFIR Frameworks: A Qualitative Study. Front Public Health [Internet]. 2020 [cited 2022 Dec 13];8. Available from: https://www.frontiersin.org/articles/10.3389/fpubh.2020.00059

11. Hong QN, Pluye P, Fabregues S, Barlette G, Boardman F, Cargo M, et al. Mixed Methods Appraisal Tool (MMAT) Version 2018 User Guide [Internet]. Canadian Intellectual Property Office, Industry Canada; 2018 [cited 2022 Dec 9]. Available from: https://www.nccmt.ca/knowledge-repositories/search/232%20(accessed%20May%202017)

12. Shepherd J, Kavanagh J, Picot J, Cooper K, Harden A, Barnett-Page E, et al. The effectiveness and cost-effectiveness of behavioural interventions for the prevention of sexually transmitted infections in young people aged 13-19: a systematic review and economic evaluation. Health Technol Assess Winch Engl. 2010 Feb;14(7):1–206, iii–iv.

13. Rees R, Oliver K, Woodman J, Thomas J. The views of young children in the UK about obesity, body size, shape and weight: a systematic review. BMC Public Health. 2011 Mar 25;11(1):188.

14. Lewin S, Booth A, Glenton C, Munthe-Kaas H, Rashidian A, Wainwright M, et al. Applying GRADE-CERQual to qualitative evidence synthesis findings: introduction to the series. Implement Sci. 2018 Jan 25;13(1):2.

15. Carroll C, Booth A, Cooper K. A worked example of ‘best fit’ framework synthesis: A systematic review of views concerning the taking of some potential chemopreventive agents. BMC Med Res Methodol. 2011 Mar 16;11(1):29.

16. Thomas J, Harden A. Methods for the thematic synthesis of qualitative research in systematic reviews. BMC Med Res Methodol. 2008 Jul 10;8(1):45.
