# Supplementary material for: Barriers and facilitators to implementing workplace interventions to promote mental health: qualitative evidence synthesis
Source: Syst Rev. 2024 Jun 7;13:152. doi: 10.1186/s13643-024-02569-2 (PMC11157821; doi:10.1186/s13643-024-02569-2)
Supplement: Supplementary file 3 — Additional file 3. Search strategy. [file 13643_2024_2569_MOESM3_ESM.docx]

**Additional File 3: Search Strategies.**

**Pubmed**

**Filters:** English language; published since 2008

**Search: ((("occupational wellbeing"[tiab] OR "occupational well-being"[tiab] OR "occupational mental health"[tiab] OR "place of work"[tiab] OR worksite[tiab] OR "work-site"[tiab] OR "job-site"[tiab] OR "workplace"[tiab] OR "work-place"[tiab] OR "work-setting"[tiab] OR "work-location"[tiab]) OR (("Workplace"[Mesh]) OR "Occupational Medicine"[Mesh]) OR (("Occupational Health"[Mesh]) OR "Occupational Health Services"[Mesh]) OR ("Occupational Diseases"[Mesh:noexp]))**

**AND**

**((suicid*[tiab] OR bully*[tiab] OR stigma[tiab] OR burnout OR "burn-out" OR stress* OR depress* OR anxious OR anxiety[tiab] OR panic[tiab] OR mood disorder*[tiab] OR wellbeing[tiab] OR "well-being"[tiab]) ORdepre (((((("Mood Disorders"[Mesh]) OR "Somatoform Disorders"[Mesh]) OR "Anxiety Disorders"[Mesh]) OR "Mental Health"[Mesh]) OR "Stress, Psychological"[Mesh]) OR "Mental Fatigue"[Mesh]) OR ((((("Anxiety"[Mesh]) OR "Psychological Distress"[Mesh]) OR "Depression"[Mesh]) OR "Bullying"[Mesh]) OR "Self-Injurious Behavior"[Mesh]) OR ("mental health"[tiab] OR "mental illness"[tiab] OR "mental wellbeing"[tiab] OR "mental well-being"[tiab]))**

**AND**

**((training*[tiab] OR campaign*[tiab] OR awareness[tiab] OR initiative*[tiab] OR policies[tiab] OR policy[tiab] OR program*[tiab] OR programme*[tiab] OR intervention*[tiab] OR service*[tiab]) OR ((("Mental Health Services"[Mesh]) OR "Occupational Health Services"[Mesh]) OR "Preventive Health Services"[Mesh:noexp]) OR ((((((("Consumer Health Information"[Mesh]) OR "Health Education"[Mesh:noexp]) OR "Patient Education as Topic"[Mesh:noexp]) OR "Primary Prevention"[Mesh:noexp]) OR "Secondary Prevention"[Mesh]) OR "Tertiary Prevention"[Mesh]) OR "Internet-Based Intervention"[Mesh]))**

**AND**

**(("evidence into practice"[tiab] OR "quality improvement"[tiab] OR reach[tiab] OR fidelity[tiab] OR "RE-AIM"[tiab] OR reaim[tiab] OR barrier*[tiab] OR facilitator*[tiab] OR acceptab*[tiab] OR adherence[tiab] OR compliance[tiab] OR implement*[tiab] OR pilot[tiab] OR feasibil*[tiab]) OR ("process evaluation"[tiab] OR "process factor*"[tiab] OR "process assessment"[tiab] OR "process outcome*"[tiab] OR "process change*"[tiab] OR "process theor*"[tiab]) OR ("change model"[tiab] OR "programme model"[tiab] OR "program model"[tiab] OR "failure model"[tiab] OR "logic model"[tiab] OR "change theory"[tiab] OR "change theories"[tiab] OR "programme theory"[tiab] OR "programme theories"[tiab] OR "program theory"[tiab] OR "program theories"[tiab] OR "failure theory"[tiab] OR "failure theories"[tiab]) OR ("research translat*"[tiab] OR "research transfer*"[tiab] OR "research use"[tiab] OR "research utilisation"[tiab] OR "research utilization"[tiab] OR "utilise research"[tiab] OR "utilize research"[tiab] OR "research disseminat*"[tiab] OR "research uptake"[tiab] OR "uptake of research"[tiab]) OR ("knowledge translat*"[tiab] OR "knowledge transfer*"[tiab] OR "knowledge use"[tiab] OR "knowledge utilisation"[tiab] OR "knowledge utilization"[tiab] OR "utilise knowledge"[tiab] OR "utilize knowledge"[tiab] OR "knowledge disseminat*"[tiab] OR "knowledge uptake"[tiab] OR "uptake of knowledge"[tiab]) OR ("evidence translat*"[tiab] OR "evidence transfer*"[tiab] OR "evidence use"[tiab] OR "evidence utilisation"[tiab] OR "evidence utilization"[tiab] OR "utilise evidence"[tiab] OR "evidence knowledge"[tiab] OR "evidence disseminat*"[tiab] OR "evidence uptake"[tiab] OR "uptake of evidence"[tiab]) OR (((((((((("Diffusion of Innovation"[Mesh]) OR "Quality Assurance, Health Care"[Mesh:noexp]) OR "Guideline Adherence"[Mesh]) OR "Process Assessment, Health Care"[Mesh]) OR "Health Plan Implementation"[Mesh]) OR "Implementation Science"[Mesh]) OR "Quality Indicators, Health Care"[Mesh]) OR "Quality Improvement"[Mesh]) OR "Capacity Building"[Mesh]) OR "Translational Medical Research"[Mesh])) AND ( "2008/01/01"[PDat] : "2022/08/18"[PDat] )**

**AND Humans[Mesh]**

**Scopus**

**Filters:** English language; published since 2008

**Search:**

( TITLE-ABS-KEY ( {occupational wellbeing}  OR  {occupational well-being}  OR  {occupational mental health}  OR  {place of work}  OR  {worksite}  OR  {work-site}  OR  {job-site}  OR  {workplace}  OR  {work-place}  OR  {work-setting}  OR  {work-location} ) )  AND  ( TITLE-ABS-KEY ( {training*}  OR  {campaign*}  OR  {awareness}  OR  {initiative*}  OR  {policies}  OR  {policy}  OR  {program*}  OR  {programme*}  OR  {intervention*}  OR  {service*} ) )  AND  ( ( TITLE-ABS-KEY ( {suicid*}  OR  {bully*}  OR  {stigma*}  OR  {burnout}  OR  "burn- out"  OR  {stress*}  OR  {depress*}  OR  {anxious}  OR  {anxiety}  OR  {panic}  OR  {mood disorder*}  OR  {wellbeing}  OR  "well-being" ) )  OR  ( TITLE-ABS-KEY ( "mental health"  OR  "mental illness"  OR  "mental wellbeing"  OR  "mental well-being" ) ) )  AND  ( ( TITLE-ABS-KEY ( ( evidence  W/2  translat* )  OR  ( evidence  AND w/2transfer* )  OR  ( evidence  W/2  use )  OR  ( evidence  W/2  utili?ation )  OR  ( evidence  W/2  utili?e )  OR  ( evidence  W/2  disseminat* )  OR  ( evidence  W/2  uptake ) ) )  OR  ( TITLE-ABS-KEY ( ( knowledge  W/2  translat* )  OR  ( knowledge  AND w/2transfer* )  OR  ( knowledge  W/2  use )  OR  ( knowledge  W/2  utili?ation )  OR  ( knowledge  W/2  utili?e )  OR  ( knowledge  W/2  disseminat* )  OR  ( knowledge  W/2  uptake ) ) )  OR  ( TITLE-ABS-KEY ( ( research  W/2  translat* )  OR  ( research  AND w/2transfer* )  OR  ( research  W/2  use )  OR  ( research  W/2  utili?ation )  OR  ( research  W/2  utili?e )  OR  ( research  W/2  disseminat* )  OR  ( research  W/2  uptake ) ) )  OR  ( TITLE-ABS-KEY ( ( theor*  W/2  change )  OR  ( theor*  W/2  programme )  OR  ( theor*  W/2  program )  OR  ( theor*  W/2  failure ) ) )  OR  ( TITLE-ABS-KEY ( ( model  W/1  change )  OR  ( model  W/1  programme )  OR  ( model  W/1  program )  OR  ( model  W/1  failure )  OR  ( model  W/1  logic ) ) )  OR  ( TITLE-ABS-KEY ( ( process  PRE/1  evaluation* )  OR  ( process  PRE/1  factor* )  OR  ( process  PRE/1  assessment* )  OR  ( process  PRE/1  outcome* )  OR  ( process  PRE/1  change* )  OR  ( process  PRE/1  theor* ) ) )  OR  ( TITLE-ABS-KEY ( "evidence into practice"  OR  "quality improvement"  OR  {reach}  OR  {fidelity}  OR  {RE-AIM}  OR  {reaim}  OR  {barrier*}  OR  {facilitator*}  OR  {acceptab*}  OR  {adherence}  OR  {compliance}  OR  {implement*}  OR  {pilot}  OR  {feasibil*} ) ) )

**Web of Science**

1. **(("place of work" OR "occupational mental health" OR “occupational well-being” OR "occupational wellbeing" OR "job site" OR worksite OR "work site" OR workplace OR "work place" OR workplace OR "work setting" OR "work location"))**
2. **(suicid* OR bully* OR stigma* OR burnout* OR "burn out" OR stress* OR depress* OR anxiet* OR anxious* OR panic OR "mood disorder*" OR "mental health") OR TOPIC: ((mental NEAR/0 wellbeing)) OR TOPIC: ((mental NEAR/0 “well being”)) OR TOPIC: ((mental NEAR/0 illness)) OR TOPIC: ((mental NEAR/0 disorder*))**
3. **(training* OR campaign* OR awareness* OR initiative* OR policies OR policy OR program* OR prevent* OR promot* OR interven* OR service*)**
4. **((knowledge NEAR/2 translat*) OR (knowledge NEAR /2transfer*) OR (knowledge NEAR /2 use) OR (knowledge NEAR /2 utili?ation) OR (knowledge NEAR /2 utili?e) OR (knowledge NEAR /2 disseminat*) OR (knowledge NEAR /2 uptake)) OR TOPIC: ((evidence NEAR/2 translat*) OR (evidence NEAR /2transfer*) OR (evidence NEAR /2 use) OR (evidence NEAR /2 utili?ation) OR (evidence NEAR /2 utili?e) OR (evidence NEAR /2 disseminat*) OR (evidence NEAR /2 uptake)) OR TOPIC: ((research NEAR/2 translat*) OR (research NEAR /2transfer*) OR (research NEAR /2 use) OR (research NEAR /2 utili?ation) OR (research NEAR /2 utili?e) OR (research NEAR /2 disseminat*) OR (research NEAR /2 uptake)) OR TOPIC: ("quality improvement" OR "evidence into practice" OR reach* OR fidelity OR "RE-AIM" OR REAIM OR barrier* OR facilitator* OR acceptab* OR adhere* OR compliance* OR implement* OR pilot* OR feasibil*) OR TOPIC: ((theor* NEAR/1 change) OR (theor* NEAR/0 programme) OR (theor* NEAR/0 program) OR (theor* NEAR/0 failure)) OR TOPIC: ((model NEAR/1 change) OR (model NEAR/0 programme) OR (model NEAR/0 program) OR (model NEAR/0 failure) OR (model NEAR/0 logic)) OR TOPIC: ((process NEAR/0 evaluation*) OR (process NEAR/1 factor*) OR (process NEAR/1 assessment*) OR (process NEAR/0 outcome*) OR (process NEAR/1 change*) OR (process NEAR/1 theor*))**
5. **#4 AND #3 AND #2 AND #1 LANGUAGES: (ENGLISH) and 2008 or 2009 or 2010 or 2011 or 2012 or 2013 or 2014 or 2015 or 2016 or 2017 or 2018 or 2019 or 2020 or 2021 or 2022 (Publication Years)**

**PsycINFO (EBSCO Host)**

| **#** | **Query** | **Limiters/Expanders** |
| --- | --- | --- |
| S92 | S91 AND S64 AND S39 AND S12 | Limiters - Publication Year: 2008-2022; English; Age Groups: Adulthood (18 yrs & older), Young Adulthood (18-29 yrs), Thirties (30-39 yrs), Middle Age (40-64 yrs); Population Group: Human Expanders - Apply equivalent subjects Search modes - Boolean/Phrase |
| S91 | S65 OR S66 OR S67 OR S68 OR S69 OR S70 OR S71 OR S72 OR S73 OR S74 OR S75 OR S76 OR S77 OR S78 OR S79 OR S80 OR S81 OR S82 OR S83 OR S84 OR S85 OR S86 OR S87 OR S88 OR S89 OR S90 | Expanders - Apply equivalent subjects Search modes - Boolean/Phrase |
| S90 | (TI model N1 (change OR programme OR program OR failure OR logic)) OR (AB model N1 (change OR programme OR program OR failure OR logic)) OR (KW model N1 (change OR programme OR program OR failure OR logic)) | Expanders - Apply equivalent subjects Search modes - Boolean/Phrase |
| S89 | (TI theor* N2 (change OR programme OR program OR failure)) OR (AB theor* N2 (change OR programme OR program OR failure)) OR (KW theor* N2 (change OR programme OR program OR failure)) | Expanders - Apply equivalent subjects Search modes - Boolean/Phrase |
| S88 | (TI process W1 (evaluation* OR factor* OR assessment* OR outcome* OR change* OR theor*)) OR (AB process W1 (evaluation* OR factor* OR assessment* OR outcome* OR change* OR theor*)) OR (KW process W1 (evaluation* OR factor* OR assessment* OR outcome* OR change* OR theor*)) | Expanders - Apply equivalent subjects Search modes - Boolean/Phrase |
| S87 | (TI feasibil*) OR (AB feasibil*) OR (KW feasibil*) | Expanders - Apply equivalent subjects Search modes - Boolean/Phrase |
| S86 | (TI pilot) OR (AB pilot) OR (KW pilot) | Expanders - Apply equivalent subjects Search modes - Boolean/Phrase |
| S85 | (TI implement*) OR (AB implement*) OR (KW implement*) | Expanders - Apply equivalent subjects Search modes - Boolean/Phrase |
| S84 | (TI compliance) OR (AB compliance) OR (KW compliance) | Expanders - Apply equivalent subjects Search modes - Boolean/Phrase |
| S83 | (TI adherence) OR (AB adherence) OR (KW adherence) | Expanders - Apply equivalent subjects Search modes - Boolean/Phrase |
| S82 | (TI acceptab*) OR (AB acceptab*) OR (KW acceptab*) | Expanders - Apply equivalent subjects Search modes - Boolean/Phrase |
| S81 | (TI facilitator*) OR (AB facilitator*) OR (KW facilitator*) | Expanders - Apply equivalent subjects Search modes - Boolean/Phrase |
| S80 | (TI barrier*) OR (AB barrier*) OR (KW barrier*) | Expanders - Apply equivalent subjects Search modes - Boolean/Phrase |
| S79 | (TI "barrier* and facilitator*") OR (AB "barrier* and facilitator*") OR (KW "barrier* and facilitator*") | Expanders - Apply equivalent subjects Search modes - Boolean/Phrase |
| S78 | (TI "RE-AIM" OR TI REAIM) OR (AB "RE-AIM" OR AB REAIM) OR (KW "RE-AIM" OR KW REAIM) | Expanders - Apply equivalent subjects Search modes - Boolean/Phrase |
| S77 | (TI fidelity) OR (AB fidelity) OR (KW fidelity) | Expanders - Apply equivalent subjects Search modes - Boolean/Phrase |
| S76 | (TI reach) OR (AB reach) OR (KW reach) | Expanders - Apply equivalent subjects Search modes - Boolean/Phrase |
| S75 | (TI "quality improvement*") OR (AB "quality improvement*") OR (KW "quality improvement*") | Expanders - Apply equivalent subjects Search modes - Boolean/Phrase |
| S74 | (TI "evidence into practice") OR (AB "evidence into practice") OR (KW "evidence into practice") | Expanders - Apply equivalent subjects Search modes - Boolean/Phrase |
| S73 | (TI knowledge N2 (translat* OR transfer* OR use OR ultili?ation OR utili?e OR disseminate* OR uptake)) OR (AB knowledge N2 (translat* OR transfer* OR use OR ultili?ation OR utili?e OR disseminate* OR uptake)) OR (KW knowledge N2 (translat* OR transfer* OR use OR ultili?ation OR utili?e OR disseminate* OR uptake)) | Expanders - Apply equivalent subjects Search modes - Boolean/Phrase |
| S72 | (TI evidence N2 (translat* OR transfer* OR use OR ultili?ation OR utili?e OR disseminate* OR uptake)) OR (AB evidence N2 (translat* OR transfer* OR use OR ultili?ation OR utili?e OR disseminate* OR uptake)) OR (KW evidence N2 (translat* OR transfer* OR use OR ultili?ation OR utili?e OR disseminate* OR uptake)) | Expanders - Apply equivalent subjects Search modes - Boolean/Phrase |
| S71 | (TI research N2 (translat* OR transfer* OR use OR ultili?ation OR utili?e OR disseminate* OR uptake)) OR (AB research N2 (translat* OR transfer* OR use OR ultili?ation OR utili?e OR disseminate* OR uptake)) OR (KW research N2 (translat* OR transfer* OR use OR ultili?ation OR utili?e OR disseminate* OR uptake)) | Expanders - Apply equivalent subjects Search modes - Boolean/Phrase |
| S70 | DE "Organizational Development" | Expanders - Apply equivalent subjects Search modes - Boolean/Phrase |
| S69 | DE "Evidence Based Practice" | Expanders - Apply equivalent subjects Search modes - Boolean/Phrase |
| S68 | DE "Innovation" | Expanders - Apply equivalent subjects Search modes - Boolean/Phrase |
| S67 | DE "Knowledge Transfer" | Expanders - Apply equivalent subjects Search modes - Boolean/Phrase |
| S66 | DE "Adjustment" | Expanders - Apply equivalent subjects Search modes - Boolean/Phrase |
| S65 | DE "Compliance" OR DE "Treatment Compliance" | Expanders - Apply equivalent subjects Search modes - Boolean/Phrase |
| S64 | S42 OR S43 OR S44 OR S45 OR S46 OR S47 OR S48 OR S49 OR S50 OR S51 OR S52 OR S53 OR S54 OR S55 OR S56 OR S57 OR S58 OR S59 OR S60 OR S61 OR S62 OR S63 | Expanders - Apply equivalent subjects Search modes - Boolean/Phrase |
| S63 | (TI service*) OR (AB service*) OR (KW service*) | Expanders - Apply equivalent subjects Search modes - Boolean/Phrase |
| S62 | (TI intervention*) OR (AB intervention*) OR (KW intervention*) | Expanders - Apply equivalent subjects Search modes - Boolean/Phrase |
| S61 | (TI programme* OR TI program*) OR (AB programme* OR AB program*) OR (KW programme* OR KW program*) | Expanders - Apply equivalent subjects Search modes - Boolean/Phrase |
| S60 | (TI policies OR TI policy) OR (AB policies OR AB policy) OR (KW policies OR KW policy) | Expanders - Apply equivalent subjects Search modes - Boolean/Phrase |
| S59 | (TI initiative*) OR (AB initiative*) OR (KW initiative*) | Expanders - Apply equivalent subjects Search modes - Boolean/Phrase |
| S58 | (TI awareness) OR (AB awareness) OR (KW awareness) | Expanders - Apply equivalent subjects Search modes - Boolean/Phrase |
| S57 | (TI campaign*) OR (AB campaign*) OR (KW campaign*) | Expanders - Apply equivalent subjects Search modes - Boolean/Phrase |
| S56 | (TI training*) OR (AB training*) OR (KW training*) | Expanders - Apply equivalent subjects Search modes - Boolean/Phrase |
| S55 | DE "Health Care Policy" | Expanders - Apply equivalent subjects Search modes - Boolean/Phrase |
| S54 | DE "Mental Health Programs" | Expanders - Apply equivalent subjects Search modes - Boolean/Phrase |
| S53 | DE "Mental Health Services" | Expanders - Apply equivalent subjects Search modes - Boolean/Phrase |
| S52 | DE "Anxiety Management" | Expanders - Apply equivalent subjects Search modes - Boolean/Phrase |
| S51 | DE "Stress Management" | Expanders - Apply equivalent subjects Search modes - Boolean/Phrase |
| S50 | DE "Health Promotion" | Expanders - Apply equivalent subjects Search modes - Boolean/Phrase |
| S49 | DE "Suicide Prevention" | Expanders - Apply equivalent subjects Search modes - Boolean/Phrase |
| S48 | DE "Relapse Prevention" | Expanders - Apply equivalent subjects Search modes - Boolean/Phrase |
| S47 | DE "Primary Mental Health Prevention" | Expanders - Apply equivalent subjects Search modes - Boolean/Phrase |
| S46 | DE "Preventive Medicine" | Expanders - Apply equivalent subjects Search modes - Boolean/Phrase |
| S45 | DE "Prevention" | Expanders - Apply equivalent subjects Search modes - Boolean/Phrase |
| S44 | DE "Early Intervention" | Expanders - Apply equivalent subjects Search modes - Boolean/Phrase |
| S43 | DE "Intervention" | Expanders - Apply equivalent subjects Search modes - Boolean/Phrase |
| S42 | DE "Treatment" | Expanders - Apply equivalent subjects Search modes - Boolean/Phrase |
| S41 | DE "Mental Health Literacy" | Expanders - Apply equivalent subjects Search modes - Boolean/Phrase |
| S40 | DE "Workplace Intervention" | Expanders - Apply equivalent subjects Search modes - Boolean/Phrase |
| S39 | S13 OR S14 OR S15 OR S16 OR S17 OR S18 OR S19 OR S20 OR S21 OR S22 OR S23 OR S24 OR S25 OR S26 OR S27 OR S28 OR S29 OR S30 OR S31 OR S32 OR S33 OR S34 OR S35 OR S36 OR S37 OR S38 OR S41 | Expanders - Apply equivalent subjects Search modes - Boolean/Phrase |
| S38 | (TI wellbeing OR TI "well-being") OR (AB wellbeing OR AB "well-being") OR (KW wellbeing OR KW "well-being") | Expanders - Apply equivalent subjects Search modes - Boolean/Phrase |
| S37 | (TI wellbeing OR TI "well-being") OR (AB wellbeing OR AB "well-being") OR (KW wellbeing OR KW "well-being") | Expanders - Apply equivalent subjects Search modes - Boolean/Phrase |
| S36 | (TI wellbeing OR TI "well-being") OR (AB wellbeing OR AB "well-being") OR (KW wellbeing OR KW "well-being") | Expanders - Apply equivalent subjects Search modes - Boolean/Phrase |
| S35 | (TI mental W2 (health OR illness* OR wellbeing OR "well-being")) OR (AB mental W2 (health OR illness* OR wellbeing OR "well-being")) OR (KW mental W2 (health OR illness* OR wellbeing OR "well-being")) | Expanders - Apply equivalent subjects Search modes - Boolean/Phrase |
| S34 | (TI "mood disorder*") OR (AB "mood disorder*") OR (KW "mood disorder*") | Expanders - Apply equivalent subjects Search modes - Boolean/Phrase |
| S33 | (TI panic) OR (AB panic) OR (KW panic) | Expanders - Apply equivalent subjects Search modes - Boolean/Phrase |
| S32 | (TI anxiety) OR (AB anxiety) OR (KW anxiety) | Expanders - Apply equivalent subjects Search modes - Boolean/Phrase |
| S31 | (TI anxious) OR (AB anxious) OR (KW anxious) | Expanders - Apply equivalent subjects Search modes - Boolean/Phrase |
| S30 | (TI depress*) OR (AB depress*) OR (KW depress*) | Expanders - Apply equivalent subjects Search modes - Boolean/Phrase |
| S29 | (TI stress*) OR (AB stress*) OR (KW stress*) | Expanders - Apply equivalent subjects Search modes - Boolean/Phrase |
| S28 | (TI burnout OR TI "burn-out") OR (AB burnout OR AB "burn-out") OR (KW burnout OR KW "burn-out") | Expanders - Apply equivalent subjects Search modes - Boolean/Phrase |
| S27 | (TI stigma*) OR (AB stigma*) OR (KW stigma*) | Expanders - Apply equivalent subjects Search modes - Boolean/Phrase |
| S26 | (TI bully*) OR (AB bully*) OR (KW bully*) | Expanders - Apply equivalent subjects Search modes - Boolean/Phrase |
| S25 | (TI suicid*) OR (AB suicid*) OR (KW suicid*) | Expanders - Apply equivalent subjects Search modes - Boolean/Phrase |
| S24 | DE "Mental Health Stigma" | Expanders - Apply equivalent subjects Search modes - Boolean/Phrase |
| S23 | DE "Mental Illness (Attitudes Toward)" | Expanders - Apply equivalent subjects Search modes - Boolean/Phrase |
| S22 | DE "Depression (Emotion)" | Expanders - Apply equivalent subjects Search modes - Boolean/Phrase |
| S21 | DE "Anxiety Disorders" OR DE "Castration Anxiety" OR DE "Death Anxiety" OR DE "Generalized Anxiety Disorder" OR DE "Obsessive Compulsive Disorder" OR DE "Panic Attack" OR DE "Panic Disorder" OR DE "Phobias" OR DE "Separation Anxiety Disorder" OR DE "Trichotillomania" | Expanders - Apply equivalent subjects Search modes - Boolean/Phrase |
| S20 | DE "Anxiety" OR DE "Anxiety Sensitivity" OR DE "Computer Anxiety" OR DE "Health Anxiety" OR DE "Mathematics Anxiety" OR DE "Performance Anxiety" OR DE "Social Anxiety" OR DE "Speech Anxiety" OR DE "Test Anxiety" | Expanders - Apply equivalent subjects Search modes - Boolean/Phrase |
| S19 | DE "Distress" | Expanders - Apply equivalent subjects Search modes - Boolean/Phrase |
| S18 | DE "Psychological Stress" | Expanders - Apply equivalent subjects Search modes - Boolean/Phrase |
| S17 | DE "Occupational Stress" OR DE "Compassion Fatigue" | Expanders - Apply equivalent subjects Search modes - Boolean/Phrase |
| S16 | DE "Stress" | Expanders - Apply equivalent subjects Search modes - Boolean/Phrase |
| S15 | DE "Mental Health" OR DE "Mental Status" | Expanders - Apply equivalent subjects Search modes - Boolean/Phrase |
| S14 | DE "Affective Disorders" OR DE "Disruptive Mood Dysregulation Disorder" OR DE "Major Depression" OR DE "Seasonal Affective Disorder" | Expanders - Apply equivalent subjects Search modes - Boolean/Phrase |
| S13 | DE "Well Being" | Expanders - Apply equivalent subjects Search modes - Boolean/Phrase |
| S12 | S1 OR S2 OR S3 OR S4 OR S5 OR S6 OR S7 OR S8 OR S9 OR S10 OR S11 OR S40 | Expanders - Apply equivalent subjects Search modes - Boolean/Phrase |
| S11 | (TI "work-location") OR (AB "work-location") OR (KW "work-location") | Expanders - Apply equivalent subjects Search modes - Boolean/Phrase |
| S10 | (TI "work-setting") OR (AB "work-setting") OR (KW "work-setting") | Expanders - Apply equivalent subjects Search modes - Boolean/Phrase |
| S9 | (TI workplace OR TI "workplace") OR (AB workplace OR AB "workplace") OR (KW workplace OR KW "workplace") | Expanders - Apply equivalent subjects Search modes - Boolean/Phrase |
| S8 | (TI "job-site") OR (AB "job-site") OR (KW "job-site") | Expanders - Apply equivalent subjects Search modes - Boolean/Phrase |
| S7 | (TI worksite OR TI "work-site") OR (AB worksite OR AB "work-site") OR (KW worksite OR KW "work-site") | Expanders - Apply equivalent subjects Search modes - Boolean/Phrase |
| S6 | (TI "place of work") OR (AB "place of work") OR (KW "place of work") | Expanders - Apply equivalent subjects Search modes - Boolean/Phrase |
| S5 | (TI "occupational mental health") OR (AB "occupational mental health") OR (KW "occupational mental health") | Expanders - Apply equivalent subjects Search modes - Boolean/Phrase |
| S4 | (TI "occupational wellbeing" OR TI "occupational well-being") OR (AB "occupational wellbeing" OR AB "occupational well-being") OR (KW "occupational wellbeing" OR KW "occupational well-being") | Expanders - Apply equivalent subjects Search modes - Boolean/Phrase |
| S3 | DE "Working Conditions" OR DE "Job Enrichment" OR DE "Noise Levels (Work Areas)" OR DE "Occupational Safety" OR DE "Telecommuting" OR DE "Work Rest Cycles" OR DE "Work Week Length" OR DE "Workday Shifts" OR DE "Working Space" | Expanders - Apply equivalent subjects Search modes - Boolean/Phrase |
| S2 | DE "Occupational Health" OR DE "Work Related Illnesses" | Expanders - Apply equivalent subjects Search modes - Boolean/Phrase |
| S1 | DE "Occupational Health Psychology" | Expanders - Apply equivalent subjects Search modes - Boolean/Phrase |

**Cinahl (EBSCOHost)**

| **#** | **Query** | **Limiters/Expanders** |
| --- | --- | --- |
| S38 | S5 AND S14 AND S23 AND S37 | Limiters - Published Date: 20080101-20220831; English Language; Human; Age Groups: All Adult Expanders - Apply equivalent subjects Search modes - Boolean/Phrase |
| S37 | S24 OR S25 OR S26 OR S27 OR S28 OR S29 OR S30 OR S31 OR S32 OR S33 OR S34 OR S35 OR S36 | Expanders - Apply equivalent subjects Search modes - Boolean/Phrase |
| S36 | ( TI (model N1 (change OR programme OR program OR failure OR logic)) ) OR ( AB (model N1 (change OR programme OR program OR failure OR logic)) ) OR ( KW (model N1 (change OR programme OR program OR failure OR logic)) ) | Expanders - Apply equivalent subjects Search modes - Boolean/Phrase |
| S35 | ( TI (theor* N2 (change OR programme OR program OR failure)) ) OR ( AB (theor* N2 (change OR programme OR program OR failure)) ) OR ( KW (theor* N2 (change OR programme OR program OR failure)) ) | Expanders - Apply equivalent subjects Search modes - Boolean/Phrase |
| S34 | ( TI (process W1 (evaluation* OR factor* OR assessment* OR outcome* OR change* OR theor*)) ) OR ( AB (process W1 (evaluation* OR factor* OR assessment* OR outcome* OR change* OR theor*)) ) OR ( KW (process W1 (evaluation* OR factor* OR assessment* OR outcome* OR change* OR theor*)) ) | Expanders - Apply equivalent subjects Search modes - Boolean/Phrase |
| S33 | ( TI ("evidence into practice" OR "quality improvement" OR reach OR fidelity OR "RE-AIM" OR REAIM OR "barrier*" OR "facilitator*" OR "barrier* and facilitator*" OR acceptab* OR adherence OR compliance OR implement* OR pilot OR feasibil*) ) OR ( AB ("evidence into practice" OR "quality improvement" OR reach OR fidelity OR "RE-AIM" OR REAIM OR "barrier*" OR "facilitator*" OR "barrier* and facilitator*" OR acceptab* OR adherence OR compliance OR implement* OR pilot OR feasibil*) ) OR ( KW ("evidence into practice" OR "quality improvement" OR reach OR fidelity OR "RE-AIM" OR REAIM OR "barrier*" OR "facilitator*" OR "barrier* and facilitator*" OR acceptab* OR adherence OR compliance OR implement* OR pilot OR feasibil*) ) | Expanders - Apply equivalent subjects Search modes - Boolean/Phrase |
| S32 | ( TI (knowledge N2 (translat* OR transfer* OR use OR utili?ation OR utili?e OR disseminat* OR uptake)) ) OR ( AB (knowledge N2 (translat* OR transfer* OR use OR utili?ation OR utili?e OR disseminat* OR uptake)) ) OR ( KW (knowledge N2 (translat* OR transfer* OR use OR utili?ation OR utili?e OR disseminat* OR uptake)) ) | Expanders - Apply equivalent subjects Search modes - Boolean/Phrase |
| S31 | ( TI (research N2 (translat* OR transfer* OR use OR utili?ation OR utili?e OR disseminat* OR uptake)) ) OR ( AB (research N2 (translat* OR transfer* OR use OR utili?ation OR utili?e OR disseminat* OR uptake)) ) OR ( KW (research N2 (translat* OR transfer* OR use OR utili?ation OR utili?e OR disseminat* OR uptake)) ) | Expanders - Apply equivalent subjects Search modes - Boolean/Phrase |
| S30 | ( TI (evidence N2 (translat* OR transfer* OR use OR utili?ation OR utili?e OR disseminat* OR uptake)) ) OR ( AB (evidence N2 (translat* OR transfer* OR use OR utili?ation OR utili?e OR disseminat* OR uptake)) ) OR ( KW (evidence N2 (translat* OR transfer* OR use OR utili?ation OR utili?e OR disseminat* OR uptake)) ) | Expanders - Apply equivalent subjects Search modes - Boolean/Phrase |
| S29 | (MH "Guideline Adherence") | Expanders - Apply equivalent subjects Search modes - Boolean/Phrase |
| S28 | (MH "Quality Assessment+") OR (MH "Quality Improvement+") | Expanders - Apply equivalent subjects Search modes - Boolean/Phrase |
| S27 | (MH "Diffusion of Innovation+") | Expanders - Apply equivalent subjects Search modes - Boolean/Phrase |
| S26 | (MH "Process Assessment (Health Care)+") | Expanders - Apply equivalent subjects Search modes - Boolean/Phrase |
| S25 | (MH "Implementation Science") | Expanders - Apply equivalent subjects Search modes - Boolean/Phrase |
| S24 | (MH "Program Implementation") | Expanders - Apply equivalent subjects Search modes - Boolean/Phrase |
| S23 | S15 OR S16 OR S17 OR S18 OR S19 OR S20 OR S21 OR S22 | Expanders - Apply equivalent subjects Search modes - Boolean/Phrase |
| S22 | (MH "Health Literacy") | Expanders - Apply equivalent subjects Search modes - Boolean/Phrase |
| S21 | (MH "Health Promotion") | Expanders - Apply equivalent subjects Search modes - Boolean/Phrase |
| S20 | (MH "Patient Education") | Expanders - Apply equivalent subjects Search modes - Boolean/Phrase |
| S19 | (MH "Health Education+") | Expanders - Apply equivalent subjects Search modes - Boolean/Phrase |
| S18 | (MH "Preventive Health Care+") | Expanders - Apply equivalent subjects Search modes - Boolean/Phrase |
| S17 | (MH "Early Intervention") | Expanders - Apply equivalent subjects Search modes - Boolean/Phrase |
| S16 | (MH "Mental Health Services+") | Expanders - Apply equivalent subjects Search modes - Boolean/Phrase |
| S15 | ( TI (training* OR campaign* OR awareness OR initiative* OR policies OR policy OR program* OR programme* OR intervention* OR service*) ) OR ( AB (training* OR campaign* OR awareness OR initiative* OR policies OR policy OR program* OR programme* OR intervention* OR service*) ) OR ( KW (training* OR campaign* OR awareness OR initiative* OR policies OR policy OR program* OR programme* OR intervention* OR service*) ) | Expanders - Apply equivalent subjects Search modes - Boolean/Phrase |
| S14 | S6 OR S7 OR S8 OR S9 OR S10 OR S11 OR S12 OR S13 | Expanders - Apply equivalent subjects Search modes - Boolean/Phrase |
| S13 | (MH "Mental Health") | Expanders - Apply equivalent subjects Search modes - Boolean/Phrase |
| S12 | (MH "Anxiety Disorders+") | Expanders - Apply equivalent subjects Search modes - Boolean/Phrase |
| S11 | (MH "Somatoform Disorders+") | Expanders - Apply equivalent subjects Search modes - Boolean/Phrase |
| S10 | (MH "Behavioral Symptoms+") | Expanders - Apply equivalent subjects Search modes - Boolean/Phrase |
| S9 | (MH "Stress+") | Expanders - Apply equivalent subjects Search modes - Boolean/Phrase |
| S8 | (MH "Psychological Well-Being") | Expanders - Apply equivalent subjects Search modes - Boolean/Phrase |
| S7 | ( TI (mental W2 (health OR illness* OR wellbeing OR "well-being") ) OR ( AB (mental W2 (health OR illness* OR wellbeing OR "well-being") ) OR ( KW (mental W2 (health OR illness* OR wellbeing OR "well-being") ) | Expanders - Apply equivalent subjects Search modes - Boolean/Phrase |
| S6 | ( TI (suicid* OR bully* OR stigma* OR burnout OR "burn-out" OR stress* OR depress* OR anxious OR anxiety OR panic OR "mood disorder" OR wellbeing OR "well-being") ) OR ( AB (suicid* OR bully* OR stigma* OR burnout OR "burn-out" OR stress* OR depress* OR anxious OR anxiety OR panic OR "mood disorder" OR wellbeing OR "well-being") ) OR ( KW (suicid* OR bully* OR stigma* OR burnout OR "burn-out" OR stress* OR depress* OR anxious OR anxiety OR panic OR "mood disorder" OR wellbeing OR "well-being") ) | Expanders - Apply equivalent subjects Search modes - Boolean/Phrase |
| S5 | S1 OR S2 OR S3 OR S4 | Expanders - Apply equivalent subjects Search modes - Boolean/Phrase |
| S4 | (MH "Work+") | Expanders - Apply equivalent subjects Search modes - Boolean/Phrase |
| S3 | (MH "Occupational Health Services+") | Expanders - Apply equivalent subjects Search modes - Boolean/Phrase |
| S2 | (MH "Work Environment+") | Expanders - Apply equivalent subjects Search modes - Boolean/Phrase |
| S1 | ( TI ("occupational wellbeing" OR "occupational well-being" OR "occupational mental health" OR "place of work" OR worksite OR "work-site" OR "job-site" OR workplace OR "work-place" OR "work-setting" OR "work-location") ) OR ( AB ("occupational wellbeing" OR "occupational well-being" OR "occupational mental health" OR "place of work" OR worksite OR "work-site" OR "job-site" OR workplace OR "work-place" OR "work-setting" OR "work-location") ) OR ( KW ("occupational wellbeing" OR "occupational well-being" OR "occupational mental health" OR "place of work" OR worksite OR "work-site" OR "job-site" OR workplace OR "work-place" OR "work-setting" OR "work-location") ) | Expanders - Apply equivalent subjects Search modes - Boolean/Phrase |

**Google Scholar (25 page relevant)**

[www.scholar.google.com](http://www.scholar.google.com)

**Filters:** 2008-2022

**Search:** (intervention OR awareness OR initiative OR training OR policy OR policies OR program OR programme OR service) AND ("mental health" OR stress OR burnout OR depression OR anxiety OR stigma) AND (workplace OR organisation OR occupational OR work)

**IOSH**

[www.iosh.com](http://www.iosh.com)

1. ‘resources & Research’ > ‘All books and Resources’ > ‘search by keyword’:
2. ‘mental’
3. ‘wellbeing’
4. ‘stress’
5. ‘suicide’
6. ‘burnout’
7. ‘fatigue’
8. ‘depression’
9. ‘anxiety’

**PROSPERO**

[www.crd.york.ac.uk](http://www.crd.york.ac.uk)

#1 MeSH DESCRIPTOR workplace EXPLODE ALL TREES

#2 MeSH DESCRIPTOR Occupational Stress EXPLODE ALL TREES

#3 MeSH DESCRIPTOR Occupational Medicine EXPLODE ALL TREES

#4 MeSH DESCRIPTOR Occupational Health EXPLODE ALL TREES

#4 MeSH DESCRIPTOR Occupational Health Services EXPLODE ALL TREES

#5 Occupational diseases OR occupational well* OR occupational mental* OR workplace

OR job site OR work location OR organisational

#6 #1 OR #2 OR #3 OR #4 OR #5

#7 MeSH DESCRIPTOR Mental Fatigue

#8 MeSH DESCRIPTOR Bullying

#9 MeSH DESCRIPTOR Depressive Disorder EXPLODE ALL TREES

#10 MeSH DESCRIPTOR Mental Health

#11 MeSH DESCRIPTOR Occupational Stress EXPLODE ALL TREES

#12 MeSH DESCRIPTOR Mental Disorders EXPLODE ALL TREES

#13 MeSH DESCRIPTOR Anxiety Disorders EXPLODE ALL TREES

#14 MeSH DESCRIPTOR Somatoform Disorders EXPLODE ALL TREES

#15 MeSH DESCRIPTOR Mood Disorders EXPLODE ALL TREES

#16 mental *

#17 wellbeing*

#18 mood disorder*

#19 anxiety

#20 anxious

#21 depress*

#22 stress*

#23 burnout*

#24 stigma*

#25 bully*

#26 suicid*

#27 #7 OR #8 OR #9 OR #10 OR #11 OR #12 OR #13 OR #14 OR #15 OR #16 OR #17 OR #18 OR #19 OR #20 OR #21 OR #22 OR #23 OR #24 OR #25 OR #26

#28 intervention*

#29 service*

#30 program*

#31 policy

#32 policies

#33 initiative*

#34 awareness

#35 campaign*

#36 training*

#37 MeSH DESCRIPTOR Internet-Based Intervention EXPLODE ALL TREES

#38 MeSH DESCRIPTOR Tertiary Prevention EXPLODE ALL TREES

#39 MeSH DESCRIPTOR Secondary Prevention EXPLODE ALL TREES

#40 MeSH DESCRIPTOR Primary Prevention

#41 MeSH DESCRIPTOR Consumer Health Information EXPLODE ALL TREES

#42 MeSH DESCRIPTOR Patient Education as Topic EXPLODE ALL TREES

#43 MeSH DESCRIPTOR Occupational Health Services EXPLODE ALL TREES

#44 MeSH DESCRIPTOR Mental Health Services EXPLODE ALL TREES

#45 #28 OR #29 OR #30 OR #31 OR #32 OR #33 OR #34 OR #35 OR #36 OR #37 OR #38 OR #39 OR #40 OR #41 OR #42 OR #43 OR #44

#46 MeSH DESCRIPTOR Translational Medical Research EXPLODE ALL TREES

#47 MeSH DESCRIPTOR Capacity Building EXPLODE ALL TREES

#48 MeSH DESCRIPTOR Quality Improvement EXPLODE ALL TREES

#49 MeSH DESCRIPTOR Quality indicators, Health Care EXPLODE ALL TREES

#50 MeSH DESCRIPTOR Implementation Science EXPLODE ALL TREES

#51 MeSH DESCRIPTOR Health Plan Implementation EXPLODE ALL TREES

#52 MeSH DESCRIPTOR Outcome and Process Assessment, Health Care EXPLODE ALL TREES

#53 MeSH DESCRIPTOR Guideline Adherence EXPLODE ALL TREES

#54 MeSH DESCRIPTOR Quality Assurance, Healthcare EXPLODE ALL TREES

#55 MeSH DESCRIPTOR Diffusion of Innovation EXPLODE ALL TREES

#56 failure theor* OR logic model* OR program* model* OR process model* OR process theor* OR process change* OR process measure* OR process outcome* OR process assessment* OR process factor* OR process evaluation* OR implement* OR compliance OR adherence OR acceptab* OR barrier* OR facilitator* OR REAIM OR re-AIM OR fidelity OR reach OR quality improvement*

#57 #46 OR #47 OR #48 OR #49 OR #50 OR #51 OR #52 OR #53 OR #54 OR #55 OR #56

#58 #6 AND #27 AND #45 AND #56

**Campbell Collaboration**

[www.campbellcollaboration.org](http://www.campbellcollaboration.org) > ‘Research evidence’ > Full text keyword search >

‘organisational’ OR ‘mental health’ OR ‘mental wellbeing’ OR ‘depression’ OR ‘workplace’ OR ‘stress’

**HTA**

[www.inahta.org/hta-database](http://www.inahta.org/hta-database)

1. Mental health OR depression OR mental wellbeing OR anxiety OR burnout OR stigma OR stress
2. Organisational OR occupational OR workplace OR job OR organisation OR occupation
3. 2 AND 2
