# Supplementary material for: Barriers and facilitators to implementing workplace interventions to promote mental health: qualitative evidence synthesis
Source: Syst Rev. 2024 Jun 7;13:152. doi: 10.1186/s13643-024-02569-2 (PMC11157821; doi:10.1186/s13643-024-02569-2)
Supplement: Supplementary file 9 — Additional file 9. Methods used to assess confidence in the review. [file 13643_2024_2569_MOESM9_ESM.docx]

**Additional file 9.**

**Method used to assess confidence in the review findings**

GRADE-CERQual assesses confidence in the evidence, based on the following four key components:

1. Methodological limitations of included studies: the extent to which there are concerns about the design or conduct of the primary studies that contributed evidence to an individual review finding.
2. Coherence of the review finding: an assessment of how clear and cogent the fit is between the data from the primary studies and a review finding that synthesises those data. By cogent, we mean well supported or compelling.
3. Adequacy of the data contributing to a review finding: an overall determination of the degree of richness and quantity of data supporting a review finding.
4. Relevance of the included studies to the review question: the extent to which the body of evidence from the primary studies supporting a review finding is applicable to the context (perspective or population, phenomenon of interest, setting) specified in the review question.

All findings started as high confidence and were then graded down if there were important concerns regarding any of the GRADE-CERQual components. After assessing each component, we made a judgement about the overall confidence in the evidence supporting the review finding. We judged confidence as high, moderate, low, or very low. The final assessment was be based on consensus among the review authors.
