# Supplementary material for: Barriers and facilitators to implementing workplace interventions to promote mental health: qualitative evidence synthesis
Source: Syst Rev. 2024 Jun 7;13:152. doi: 10.1186/s13643-024-02569-2 (PMC11157821; doi:10.1186/s13643-024-02569-2)
Supplement: Supplementary file 10 — Additional file 10. Themes for barriers and facilitators to implementing mental health intervention in the workplace. [file 13643_2024_2569_MOESM10_ESM.docx]

**Additional File 10 – Themes for Facilitators and barriers to implementing mental health interventions**

| **Summary of evidence – RQ2** | | | | |  |
| --- | --- | --- | --- | --- | --- |
| **Finding title (and brief description)** | | **Studies contributing to review findings** | **Analytic themes (narrative descriptions)** | | **Example quotes** |
| **Intervention Design/Characteristics** | | | | |  |
| **Finding RQ2-1: Intervention content** related to relevance and tailoring to the needs of the organisation or sector context and the participants. | | (1–12) | Several studies highlighted the importance of relevant and tailored content that was well presented in facilitating employee engagement with intervention activities and materials (1,3–8,10–12) Increasing specificity and relevance of case scenarios and practical solutions tailored to the organization and day-to-day tasks was seen as favorable across several studies (4,6,11). Another study reported tailoring of the initial questionnaire to their organizational context facilitated implementation of the intervention (2). In some studies, the benefit of specificity and relevance went beyond the organization level to include personal tailoring of activities and flexibility to suit certain staff within the organization that have unique needs (e.g., field staff) (4,7).  Often this tailoring required preparation and liaison with organization leaders, risk assessments and piloting, and in-depth sector specific knowledge prior to implementation began to ensure that content was relevant to the unique needs of the organization or sector (1–3,11).  Intervention content that did not consider wider contextual factors (e.g., workplace pressure) (9) or systemic issues (e.g., with mental health services) (10) was less valued by participants. Further, there were mixed opinions about the depth of mental health content that participants wanted (10,12). For example, some participants wanted to learn about more serious mental health problems (10,12), while others wanted more time on common mental health disorders (12). | | “Most attendees scored trainers highly for the knowledge of materials, presentation skills, diversity of learning materials, communication skills, use of a range of teaching approaches and ability to keep the course focused and relevant.” (10)(p45)  "The Beyond Silence curriculum was designed to address the  unique needs of the organization and is based on pilot data from the initial qualitative phase of the project." (11)(p5)  “Some mentees felt the content could have been pitched more appropriately to acknowledge the stressful workplace pressure they regularly faced.” (9)(p551) |
| **Finding RQ-2. Flexibility and tailoring of intervention delivery** related to when, where and how the intervention was delivered. | | (1–4,6,8,11–13) | Preferences regarding aspects of intervention delivery were reported in several studies. There were positive opinions about having flexibility for the length of interventions (3,4). Another study reported being able to choose who could chair meetings facilitated implementation of the intervention (2).  There were different preferred modes of delivery reported in the evidence. Some participants preferred digital interventions because they were considered convenient and confidential, while others found this format hard to engage with, without guidance (8,12). For some of those that like digital interventions, audio recorded components provided opportunities to use the intervention at their own pace (1,13). While in-person delivery, including practical/interactive activities, were reportedly preferred by other participants (6,8,12).  However, participants in one study reported that clinical appointments were prioritised over the digital intervention, which may not have been the case for a face-to-face intervention (8). Participants in another study found intervention activities delivered in the evening difficult to engage with in the long term (11). | | “Participants described digital mental health interventions as  being convenient both in terms of accessing it at a time that is  convenient for them and at a place that is convenient for them." (8)(p4)  “The opportunity to adapt the implementation process to the company was welcomed. Even so, the project was sometimes felt to be insufficiently flexible.” (4)(p581) |
| **Finding RQ2-3: Consolidating learning and sustaining knowledge** and skills acquired during intervention related to feedback, reminders, refresher sessions and timing of intervention activities. | | (6,8,11,12,14,15) | Participants in one study reported that too much time between intervention activities was a barrier to sustaining new knowledge and suggested that having a shorter time span between intervention sessions would help them retain information (15). Participants in another study were concerned that some intervention feedback activities were owned by external intervention providers, restricting feedback in the future (14).  Participants in other studies commented on the value of communications (e.g., emails) including reminders to log in, motivational content, and feedback reports, which helped to consolidate learning (6). However, some participants did find them annoying (8).  In two other studies, refresher sessions were suggested as good strategies for sustaining the intervention, would maintain motivation and ‘level up’ mental health promotion with physical health promotion, although these had not been part of the actual study (11,12). | | “In addition to using the different reminders withing the intervention, some participants described setting their own reminders by putting tasks in their work calendar. They noted that this helped them to engage with the program.” (8)(p6)    “They (participants) felt that refresher sessions could provide an opportunity to try out different strategies and maintain their motivation to apply the principles in their day-to-day work" (11)(p838) |
| **Finding RQ2-4 Fostering relationships, openness, and confidentiality** related to shared language and experiences with credible and relatable instructors, and composition of a participant group. | | (2,7,10–12,14–19) | Several factors relating to fostering good relationships and openness were reported in several studies (2,7,10–12,14–19).  Many studies identified the positive impact of the shared experience of participating the intervention together (6,12,17,18), which can help promote openness (18) and long-term changes culture (17). An additional study highlighted that the intervention allowed employees to get to know each other, encouraging changes in the psychosocial work environment (15).  Instructors perceived as credible and relatable also facilitated openness (7,10,12,18,19), and this could be enhanced by having trainers with a lived experience of mental health (12,18) and/or with experience of the sector (18). Conversely, instructors lacking compassion for participants with lived experiences of mental health was a barrier (12).  There were mixed opinions regarding the professional composition of group interventions, whereby, some participants preferred to be trained within in groups with mixed levels of seniority (11,18), however, others argued that this limited the opportunity to be open and wanted to be trained within peer groups or with staff of similar levels (18).  There were different preferences for internal or external intervention providers (7,10,16). While some participants in one study said that they would prefer to approach employees’ mental health first aiders they knew and trusted, others raised concerns about approaching someone who they perceived to be too close, in fear of potential confidentiality breaches (12). In some cases, internal support was seen as a barrier to engaging (10), and the perceived benefit of external providers was anonymity (7) or impartiality (16).  The importance of language in the interventions was highlighted in several studies as necessary to cultivate openness (15,18,19). Specifically, three studies highlighted the need for a shared language for communication about topics of mental health (15,18,19), for example with the word “officer” could create a feeling of safety (19), Further, language which is not clear was a barrier (14). | | “Interviewees discussed the mental health continuum model in particular as being helpful in facilitating more openness and dialogue, as it provided a common language for staff to discuss mental health and to talk about their experiences in a nonthreatening way.” (18)(p35S)  both schools, participants were moderately satisfied with the group sessions More specifically, most of the participants felt that everyone could freely give his or her opinion (88.9%) and that the chair listened to them.” (2)(p14)  “A number of people also cited that the personal qualities of the MHFAO are important because they need to establish a relationship and rapport with the person they are assisting. They would also need to be able to sensitively approach people, demonstrate non-judgmental listening skills and keep information confidential.” (19)(p373)  “Some respondents felt that the instructors lacked compassion and sensitivity for attendees who themselves had experienced mental health problem.” (12)(p57) |
| **Implementation strategy/process characteristics** | | | | |  |
| **Finding RQ2-5 Management/leadership buy-in and support at all levels** related to prioritisation, proactivity, and promotion of the intervention, appropriate authority, and continuous commitment, demonstrating the value placed on it. | | (1–5,10–12,14,16–21) | Leadership buy-in and support was highlighted as an important facilitating factor by several studies (1,3,4,10,11,18,21).  Specifically, leadership support reportedly helped to prioritise, promote, and present the value of the intervention (1,3,8,10,12,17). Support from leaders also gave the intervention weight (5) and helped to anchor it to facilitate cultural uptake (4,11,18). One study reported that senior and middle management buy-in was facilitated by a desire to solve a given problem (2).  It was noted that proactive and continuous leadership commitment to the intervention was facilitative (e.g. identifying problems and solutions, feedback, resource allocation, chairing steer group meetings, being exemplar in managing their own stress) (1,5,16,20). Leaders who are good at communication, had credibility (1), and collaborate to ensure continued commitment were particularly helpful during implementation.  Further, leadership support from every level (1,17), including trade unions (16), was also facilitative, while a lack of support in some levels of leadership hindered employee engagement or implementation (16,20). Participants in one study noted that managers were concerned about people with a history of mental health issues attending training, suggesting that they needed support to feel confident in encouraging all employees to engage with the intervention (12).  A lack of leadership, more generally, was also reported as a barrier to implementation. Studies suggested that where leaders did not demonstrate the intervention or attend training, a perception was created that the intervention was not valued (7,10,12,18–20). However, it was also noted that leadership buy-in could be cultivated if not initially existing. This could be done via leveraging support from other organisational champions and demonstrating the value of the intervention, e.g., using data regarding physical and psychological safety in the workplace (5,18,19). | | “Promotion by the employer gave the intervention legitimacy and gave the staff explicit permission to use it." (8)(p7)  “Across all case studies, senior management support was perceived as a key enabler to move the strategy forward and this was demonstrated in various ways, i.e., through resources allocation, chairing the stress strategy steering group or being exemplar in managing their own stress.” (5)(p4)  “Some participants reported what they felt was a tokenistic approach to teacher well-being and mental health, as opposed to genuine investment and commitment from senior leadership.” (10)(p65) |
| **Finding RQ2-6: Communication of clear, succinct, relevant, timely, information** related to the use of formal, appropriate, and varied channels, to share information both vertically and horizontally at every stage of the implementation. | | (1–4,6,9,10,12,14–19) | Communication was a key factor mentioned in many studies that impacted on implementation. Vertical and horizontal communication, that reached everyone, was seen to facilitate implementation (1,17), while a lack of communication across groups was identified as a barrier (3,14).    Specifically, communicating the value (18), the need for the intervention (1), the benefits (17), and how to access the intervention (12) facilitated implementation.  Alternatively, studies reported that a lack of communication about the intervention relevance (15), available support (3,10), intervention promotion (19), and action plans (2), hindered implementation.  Helpful methods of communication included using existing communication structures (17) and varied channels of communication rather than relying on emails (e.g. using posters, internal communications or bulletins, websites, badges, lanyards, email signatures, intranet or payslip) (9,12,14,16,19).  Regular communication at every stage of implementation (e.g., development, evaluation, progress, and feedback) (1,4,6,9,16) was also highlighted as facilitative in several studies. However, in one study reported that ‘progress outcomes’ which focused on work pace as opposed to the quality of work outputs were not valued by participants (14). | | “Almost all organisations used internal resources like noticeboards, the intranet, and posters to spread the word. MHFAOs were also promoted during relevant events. However, more experienced organisations had evidence of more structured ways MHFAOs could be identified. For example, embedded programmes mentioned MHFAOs during induction and used markers to help staff directly identify an MHFAO, such as on a sign or icon on office desks and doors.” (19)(p7)  “Creation of communication tools aimed to announce different phases and main results of the research and to maintain the interest of correctional officers for the process." (1)(p285) |
| **Finding RQ2-7. Change agents** related to a range of individuals, both internal and external who could drive or championed implementation or the intervention. | | (1,7,12,16,17,19,22) | Numerous studies highlighted the importance of specific individuals driving the change required to implement the intervention. These individuals included senior managers (22), local leaders (7), clinical managers and topic experts (17), a leader who also contributed to external health and wellbeing initiatives (19); and local champions (7,12,17) who in some cases were board members (16).  Two studies reported external change agents who facilitated implementation, including the research team (1,7) and an external intervention expert (1). | | “Having a board member as a stress champion or having both a board member and a senior manager as members of the steering group was thought to ease the project progress.” (16)(p4)  “Several participants’ statements describe the value in having this local champion articulate the “value-add” of the innovation by appealing to organizational cultural values.” (17)(p868)  “The persistence and flexibility of the Deakin research team, as well as its willingness to assist the Police Psychology Unit in the coordination and scheduling of mental health literacy training were acknowledged.” (7)(p20) |
| **Finding RQ2-8. Assistance and backing to engage in the intervention** related to support from colleagues, managers, intervention experts and technology teams for both employee-level participation and for the intervention activities themselves. | | (1,3,6,7,11,12,14,19,23) | Facilitating factors included managers who were proactive and supportive engagement of those in supervisory or management roles with the intervention materials. It was also crucial for employees to feel supported and empowered to engage fully with the intervention activities and to make changes within their working environment and practices. (e.g. through finding cover for shifts) (1,4,12,23) and the sharing of information and resources (e.g. through networking) (7,11,12).  A lack of support was reported across studies reflected many different forms such as: a lack of support from colleagues to engage with the intervention (e.g. covering time during shifts) (12,23), a lack of technical support for digital interventions (14), a lack of support from intervention experts (3), a lack of training (9,19); and a lack of support for intervention providers (6,19). | | “The majority of managers felt that their line manager had been supportive and would encourage  them to make changes. (6)(p21)  “An important facilitating factor … was an engaged first line manager, who also communicated clearly about the importance of everyone´s engagement” (14)(p12).  “The most common challenge cited by participants across all cases was providing adequate support to MHFAOs. Four MHFAOs reported feeling inadequately supported in their roles, such as experiencing a lack of follow-up after receiving their training or isolation from other MHFAOs.” (19)(p12) |
| **Finding RQ2-9. Stakeholder engagement** related to involvement in decisions, reaching consensus, and participator approaches to implementation involving key stakeholders at all levels. | | (1,2,4–7,11,14,16,17,19) | Stakeholder engagement was frequently reported as an influential factor for intervention implementation. Setting up a multidisciplinary stakeholder group (e.g. including people with influence, autonomy or expertise) facilitated intervention implementation (4,6,7,16). Engaging staff at all levels in decision making (e.g. via a participatory approach) was also valued (1,4,5,8,14,16,19). However, one study found staff a lower level of the organisation felt they had little influence, and that overall participation was less as the intervention progressed. The same study reported distant relationships and lack of trust between managers and staff hindered implementation (2).  It was also noted that structured communication to support engagement (19) and reaching consensus during decision-making and participation was beneficial to implementation, which was reportedly more challenging in larger groups (2,14). | | “Automated feedback and personal tips were appreciated, stimulating discussion and change, especially at the beginning of the project. The participants saw it as a sign of respect that they were asked to express their views and opinions.” (4)(p579)  “There was some variation between subgroups with regard to how engaged key stakeholders (unit members) were. This was due in part to group size. In some groups, everyone was engaged, and they discussed and decided everything together. This was more difficult in bigger groups.” (14)(p13) |
| **Finding RQ2-10: Participant’s choice** related to voluntary or mandatory participation in the intervention. | | (7,11,12,17) | The fact that intervention participation was voluntary was discussed in four studies. One study reported that participants appreciated voluntary participation because they the approach was respectful of their busy schedules (17).  Another study reported that leadership participation should be mandatory (11). Two studies highlighted those participants from male dominated sectors (i.e., police and construction) may benefit from mandatory participation (7,12) to increase uptake and overcome negative attitudes towards mental health related interventions (12). | | “The voluntary nature of the project, given it was research-based. As a result, uptake of the program by individual station members was limited…” (7)(p18)  “However, there were also people who thought that particular [negative] attitudes were prevalent among certain members of the workforce, which contributed to resistance… And some suggested that they might change these attitudes through [mandatorily] attending the training” (12)(p50-51) |
| **Finding RQ2-11: Clarity of roles, responsibilities and boundaries** relate to local implementation and intervention providers. | | (3,8,9,12) | A few studies referenced clarity around roles and responsibilities during implementation. Whilst establishing clear roles and responsibilities was a key facilitating factor for implementation (9), three studies described a lack of clarity regarding the roles, responsibilities or boundaries of those involved in the implementation as a barrier (e.g. intervention providers) (3,8,12). | | "Setting clear and realistic expectations of the outcomes, steps involved, and commitment required from both mentees and mentors for the mentoring component of the programme was raised as an important factor contributing to the overall success..." (9)(p997) |
| **Finding RQ2-12: Coherence with the organisations values, policies, and structures** related to ability to integrate or embed the intervention into the organisation. | | (1,2,4,6,10,13,14,16,18,19,21) | Several studies described the importance of alignment and integration with existing internal organisational policies or structures (1,4,6,10,13,14,16,18,19,21). Of those, one study described an organisation in which safety policies had changed to aid intervention implementation (19) and another explained that the intervention had been integrated into existing organisational systems and policies (16) and training structures (18) was then perceived to as giving mental health a clear priority and indicated a change in organisation values. perceived as giving clear priority to the stress issue.  Conversely, two other studies found a lack of integration a barrier to implementation. For example, participants in one study noted that the intervention was dramatically different from existing policy that specify no set breaks, which acted as a barrier to engagement in the intervention due to mixed messages (21). Participants in the other study reported that the intervention had not been embedded into the organisation causing frustrations that the intervention was a temporary exercise, as opposed to a serious attempt to tackle mental health (10). Another study reported that the existing hierarchical structure required to be addressed (1).  The participants in another study perceived the structured, target-driven way of implementing the intervention activities was not suited to the school structure that valued autonomy of the individual teacher (2).  Two studies made suggestions: first that the intervention should be integrated into existing organisational initiatives/policies/training structures from initiation to aid implementation (6), and second, that the intervention should continue to be integrated into training structure to sustain the intervention organisation wide (18).  Finally, one other study, including multiple organisations that chose a department- or organisation-wide implementation approach, found that smaller scale implementation allowed for local flexibility, while larger scale implementation allowed for consistency (16). | | “interviewee’s commonly noted that the existing training structures within police organizations make a program such as R2MR relatively straightforward to implement. With mandatory training blocks already in place, many organizations incorporated R2MR into this structure, enabling the program to be delivered straightforwardly in a widespread and timely manner.” (18)(p34S)  “Integration of stress policy into corporate plans and internal systems and procedures (human resources, etc.) was perceived as giving clear priority to the stress issue.” (5)(p4)  “Negative reactions and experiences were common barriers before and during the intervention. ‘I thought organized physical activity for a break at the office…was a dramatic change from our existing policy of no set breaks.” (21)(p531)  The participants perceived the structured, target-driven way of implementing the intervention activities in the second phase not suited to the school structure that valued autonomy of the individual teacher. (2)(p15) |
| **Finding RQ2-13: Intervention initiation** related to impact and strategy for wellbeing | | (2,10,12) | Three studies reported reasons for intervention initiation (10,12). These reasons included recognising the impact of mental health issues among staff (e.g., affecting performance, burnout, sickness absence, recognised risk factors) (2,10,12) and aligning with organisational strategy for wellbeing (12). | | “This high level of initial acceptability could be explained as schools having reached a ‘tipping point’, where they had become increasingly aware of the need to address staff mental health and well-being.” (10) |
| **Context** | | | | | |
| **Finding RQ2-14: Workload Demands** related to excessive and poorly managed workloads negatively impact implementation. | | (1,3,6–8,10,12–14,17,20,21) | Workplaces where workers consistently face unmanageable workloads present a significant barrier to implementation (1,3,6–8,10,12–14,17,20,21). Excessive workloads often leave employees with little to no additional capacity to engage with intervention activities. | | “"Over half of the interviewees identified the pressure of time or excessive workload as being the main reasons for not engaging with the intervention" (8)(p7) |
| **Finding RQ2-15: Internal Resources** related to organisations possessing the necessary staffing arrangements, time, physical environment, and financial resources to support the delivery of intervention activities. | | (1,4,6–10,13,14,16–21,23) | An organisation’s capacity to successfully implement mental health intervention activities were largely governed by their resources available to support the activities (1,4,6–10,13,14,16–21,23). This was manifested in several ways: adequate staffing (across all tiers of an organisation with consistent, adequate and stable staffing levels acting as a significant facilitator), time (affording the employees with the time to participate in the activities, offering flexibility within pre-existing standardised scheduling practices in a non-punitive manner), physical space (having available, adequate, and appropriate space required to facilitate the corresponding activities), and financial flexibility (being able to fund the components and materials of the interventions). | | See quotes below |
| **Finding RQ2-15a: *Internal Resources: Staffing***  Related to staffing across all levels of an organisation which had the potential to influence implementation of interventions. | | (1,4,5,7,14,20) | Staff levels across all tiers of an organisation can influence the implementation of interventions (1,4,5,7,14,20). Consistent, adequate and stable staffing levels at both employee and leader levels bears significant impact on the ability of an organisation to successfully implement mental health interventions (1,4,5,7,14,20). | | “Organizational barriers result from the given working conditions of clinical care and … include aspects such as staff shortages, unscheduled staff absences” (20)(p6) |
| **Finding RQ2-15b: *Internal Resources: Time*** related to organisations affording participants in intervention activities with the time to participate in the activities, and flexibility within pre-existing standardised scheduling practices. | | (1,6,7,9,10,13,16,18,19,21,23) | Organisations need to afford participants in intervention activities with the time to participate in the activities, offering flexibility within pre-existing standardised scheduling practices in a non-punitive manner (1,6,7,9,10,13,16,18,19,21,23). Intervention activities that are convenient for organisations to deliver and employees to engage with facilitate implementation. Ability for participant to plan time for intervention also facilitates engagement.  With regards to timing, an accurate estimation of the time required to achieve anticipated outcomes is also important (1,7). Moreover, participants also appreciated an estimation of the time required to follow the intervention and that they could track their progress (8).  Having planned time to take a break to engage with the intervention was seen as beneficial (21). | | “schedule/time constraints and interruption of workflow” (21) (p531)  "’Well-suited - good timing’ … ‘I decided to participate in the intervention because the schedule suited me very well’" (13)(p15) |
| **Finding RQ2-15c: *Internal Resources: Physical environment*** related to the availability and willingness of the organisations to dedicate adequate and appropriate space to meet the needs of the intervention. | | (8–10,17,23) | Timely and predictable access to adequate and appropriate space fit to the needs of the intervention activities is critical (8–10,17,23). Proximity and convenience of access for participants is viewed as a facilitating factor. | | “Finding a suitable venue for mentoring sessions, particularly when mentee–mentor partnerships were based at different sites, was seen as problematic” (9)(p996)  "’Well-suited - company facilities’… ‘the fact that it was held at the company’s facilities and during working hours was very valuable’" (13)(p15) |
| **Finding RQ2-15d: *Internal Resources: Financial*** related to organisations possessing the necessary financial resources to support the intervention. | | (1,7,17) | The organisation must be prepared and capable of committing to funding the necessary components and materials of the interventions (1,7,17). | | '"Financial imperatives often remained a very difficult obstacle to overcome." (1)(p206)  “The high cost or feasibility associated with implementing such a program state-wide. This is very resource-intensive, certainly in its current delivery style, to have all these days and meetings and workshops, it’s unusually resource-intensive.” (7)(p26) |
| **Finding RQ2-16: Organisational Change/Stability** related to significant change or transitions within an organisation. | | (2,5,7,16) | Participants within organisations undergoing periods of significant change or transition viewed this process as being a barrier to the implementation of mental health promotion intervention activities (2,5,7,16).  One study found increase various unexpected events e.g. curriculum changes, tuition fee reductions, staff changes, temporary contracts ended, which resulted in increased workload and negatively affected implementation (2). | | “Constant organizational changes [was] invoked as being [a] barrier to availability” (5)(p8) |
| **Finding RQ2-17: Culture Alignment** related to alignment of the culture of the organisation relative to the aims and objectives of the intervention activities. | | (1,6,7,18,21) | Compatibility between the intervention activities and the prevailing culture of the organisation was viewed as a key determinant of implementation success (1,6,7,18,21). Moreover, organisations where the prevailing culture was marked by the presence or absence of trust and respect proved to be integral (7). | | Barrier: “Members lacking respect or trust for station command or champion” (7)(p19).  Alignment: “It was the extent of cultural uptake that ultimately determined the program’s ability to facilitate broader culture change.” (18)(p325) |
| **Mental models** | | | | | |
| **Finding RQ2-18: Previous experience of mental health interventions and organisational initiatives** influenced perceptions of the intervention | **Finding RQ2-18a; Experience and awareness of mental health issues** related to level of engagement with a workplace mental health intervention. | (8,11–13,16,18,19,23) | Two studies also highlighted that previous experience of mental health issues, either personally or within the organisation, were reasons for initiating or engaging with the intervention (12,19). However, one study highlighted that some symptoms of depression may hinder engagement with the intervention (8), and one participant in another study suggested that participation in group interventions may be challenging for introverts (11).  Three studies (13,16,23) described employees who did not believe mental health issues were a problem, which hindered engagement with the intervention. However, in one of these studies these views changed for participants as they attended the intervention, thus realising its value (23). Increasing awareness of mental health issues through intervention attendance, alongside positively experiencing the intervention, was also noted in three other studies as a trigger for culture change (12,18,19). | | The nurses attending the intervention commented that they were also prejudice at the beginning but when they continued to attend the intervention their prejudices disappeared, and they expected the intervention to contribute to their individual development.” (23)(p240)  “Some interviewees indicated that their interest in MHFA originated from personal experiences and wanting to help others who might be going through a similar situation...Other interviewees did not describe a specific interest originating from personal experience; rather, they stated that their personality traits – or altruistic reasons – encouraged them to pursue training“ (12)(p51) |
|  | **Finding RQ2-18b. Experience of previous organisational initiatives.**  related to expectations and attitudes towards the intervention. | (1,15,17,23) | One study noted that positive experiences of previously using similar interventions facilitated a positive attitude and implementation of the intervention (17). Conversely, three studies highlighted those previous negative experiences of workplace interventions (e.g., which had failed or were seen as monotonous, repetitive or unable to make a change) had a negative impact on their expectations of the intervention (1,15,23). | | "Past experience with mindfulness was viewed as positively influencing attitudes toward the initiative and thus facilitating implementation." (17)(p865)  "Since the past trainings were monotonous and repetitions of the same things, we thought that this would not make any contributions. (23)(p240) |
| **Finding RQ2-19: Perception of intervention as motivation to engage** related to participants’ relatability and level of interest in workplace stress, positive reputation of the intervention perceptions of the usefulness and progress made**.** | | (2,6,10–14,17,20,21) | Two studies reported that participants had rated and perceived the usefulness of interventions (6,11). Building a positive reputation for the intervention was suggested as a facilitating factor (11). In other studies, some participants did not think the intervention was useful at all (6,10), and in some cases, the intervention was perceived to be ‘tokenistic’, rather than genuine (10).  Lack of self-discipline (20), motivation and interest (21) were highlighted by participants in studies of non-digital interventions. However, participants in two other studies of face-to-face interventions reported that curiosity about the intervention and associated study, personal responsibility and exposure to the intervention encouraged them to attend the intervention (13,17)  Moreover, while noticing progress was reported to boost participants’ willingness to continue using the intervention (14), experiences of stagnation in progress or no progress resulted in less engagement (2,14)  . | | “Although the positive effects of breaks and finishing work on time [used as stress prevention measures for leaders] are known, they were hindered, e.g., by the discipline of the individual…” (20)(p6)  “The majority of participants who attended the training were enthusiastic about the MHFA training courses and their potential value. Most participants were also positive about the peer support service in principle“ (10)(p63) |
| **Finding RQ2-20:** **Stigma about mental health and perceived confidentiality issues** related to participants willingness to engage in the intervention, be open and talk about their experiences or gain insight into mental health problems | | (7,9–12,19,23) | Several studies mentioned stigma regarding mental health issue. Two studies highlighted the importance of sharing personal stories of mental health struggles (11,12), particularly from leaders (11), to tackle stigma and encourage open dialogue mental health. Sharing personal stories were reported to, increase insights into the mental health problems of colleagues, instil a ‘passion’ toward addressing mental health problems, and challenge the stigma associated with mental illness (11,12).  Participants in one study noted that mental health stigma was an issue in the wider organisational culture, leaving them concerned about confidentiality and reluctant to be open about any issues (10). Participants in other studies also reported being reluctant or unwilling to be open about personal issues or attend the intervention (7,9,23) due to fears of being stigmatised as being ‘unable to cope’ (9,23). It should be noted that this perception shifted during the intervention in one study (9). | | “A number of people also cited that the personal qualities of the MHFAO are important because they need to establish a relationship and rapport with the person they are assisting. They would also need to be able to sensitively approach people, demonstrate non-judgmental listening skills and keep information confidential.” (19)(p373)  “Some mentees expressed an initial reluctance to being involved with the programme related to negative connotations of the word ‘resilience’ and a misunderstanding of the course purpose prior to it commencing. I thought I was put on the Resilience Course because my manager didn’t think I was resilient enough and that it was a shortfall in my performance. (Mentee, Cohort 2). However, once the programme was completed this perspective often shifted.” (9)(p515) |
| **Summary of evidence – RQ3a Healthcare** | | | | |  |
| **Main Findings** | | **Studies contributing to review findings** | **Narrative descriptions** | | **Example Quotes** |
| **Intervention Design** | | | | | |
| **Finding RQ3a-1: Intervention content** related to relevance and tailoring to the needs of the organisation or sector context and the participants. | | (11,14,22,24–26) | The relevance and/or tailoring of intervention content was mentioned in several studies. The value of intervention content that was relevant to specific workplaces was highlighted in two studies (11,25). Adapting and customising the content and timing of the intervention to fit individual’s and organisation’s needs and abilities (e.g., via co-development or a participatory approach) was also reported as facilitative in several studies (11,14,22,24–26). | | "Some liked the structure of reviewing each type of illness or ‘disease process;’ others, particularly those with a clinical background, indicated that the information was a refresher’ about various mental health conditions. A few clinicians complained that the information was too basic with limited opportunity for new learning." (11)(p838)  "The recovery models were customized, which means that the activity range was adjusted to each PHCC’s own abilities, needs, and wishes." (26)(p5)  "Another consistent comment from the MHFA participants was that they would like more examples and case scenarios that were relevant to their workplace." (11)(p838) |
| **Finding RQ3a-2: Flexibility and tailoring of intervention delivery** related to when, where and how the intervention was delivered. | | (3,11,14,17,22,24–29) | Flexibility regarding when, where and how much of the intervention could be used was valued, whether the intervention was delivered digitally or face-to-face (3,22,25,26,28,29).  Such flexibility was conducive to the intervention being relevant for individual’s needs and their schedule (22,24,26,28,29).  Overall, short (26,28,29); and simple activities that fit with usual daily routines were seen as facilitative (26,28,29). Conversely, intervention activities that were difficult to master (22) and did not fit with daily work routine acted as a barrier (e.g., computer based activities where working on a computer is not a core task for participants) (27). App-based interventions were valued in one study because they helped fit into daily routines, however, it should also be noted that individual discipline was also seen as a barrier for digital interventions (29).  Finally, two studies reported that participation with the intervention was voluntary (11,17). Staff reported the value of not feeling coerced to use the intervention and that the time pressures that they face were respected in one study (17), while in the other, participants believed the intervention should be integrated into employee orientation (11). | | "some staff may prefer to exercise a degree of autonomy over the timing of when OH engagement (e.g., referral) should take place and this appears to be closely linked to the severity of their condition at a particular point in the sickness absence episode" (22)(p61)  "Some participants recalled using the apps on a regularly scheduled basis, and others used the apps on challenging days to manage perceived stress." (29)(p384) |
| **Finding RQ3a-3: Accessibility of the intervention** related to time required, external ownership of intervention components, availability of intervention providers, and when and where the interventions were delivered | | (3,11,14,22,25,26,28–30) | Several barriers were reported which hinder accessibility. First, there was concern that ownership of some intervention components was external (14). Two studies reported that time-consuming interventions (14,28) hindered accessibility. Interventions delivered out with usual working hours (11) or usual place of work (30) were also identified as barriers to intervention accessibility. In terms of digital interventions, technical difficulties (22,29) or lack of experience with technology (29) hindered accessibility.  Participant’s capabilities or existing knowledge also affected accessibility. Two studies found different preferences for the level of detail regarding the intervention content (11,22), which was driven by participants existing knowledge and skills gained from their professional backgrounds. Further, in another study, participants reported having difficulty interpreting data produced by the intervention’s web-based support, which provided feedback about their progress, (14), suggesting that participant’s lacked capabilities required for the intervention.  Rapid deployment of interventions, as well as visible and available intervention providers, helped participants access the intervention (25), while stunted deployment of intervention components was described as frustrating (3). | | “Some attendees struggled because the unit was not their main base.” (30)(p948)  “All of the participants affirmed that ease-of-access of PFA was an important factor in encouraging participation. This is reflected in the availability of PFA providers immediately after the TE, the visibility of PFA providers (e.g., wearing a distinctive sign from other EMS workers), or even in advertising by the organization” (25)(p952)  “it was difficult to commit to evening meetings over a 3-month period.” (11)(p838) |
| **Finding RQ3a-4: Fostering relationships, and** openness related to shared language and experiences with credible and relatable instructors and, where relevant, composition of a participant group. | | (9,11,14,17,22,25,28) | Opportunities for open dialogue and shared reflection in the group was valued (11,28) and seen to encourage openness (25) and reduce isolation (28). Specifically, participants in one study described attending training together which created a shared experience and assisted culture change (17), while participants in another study appreciated being able to share common meanings of their experience (25). Within group formats, one study highlighted that it was valuable to hear view from diverse groups of staff (i.e., unions, frontline staff and managers (11), however, there was also concern about being open in a group setting, e.g. divulging work problems or emotional difficulties in a group made up of mixed seniority (28). In two other studies, cohorts from the same services/professions were viewed positively (9,14) and reportedly facilitated a shared understanding of issues (9)  Where interventions involved a one-to-one component, a good relationship with the intervention provider was noted to promote trust and engagement (25). Participants noted that enough time (9,22) and a flexible approach to establishing goals was required to develop positive relationships (9), and that the opportunity to discuss issues in a supportive environment was seen positively (22). Furthermore, characteristics of the intervention provider, such as competency and being a good listener, were facilitative (25). | | “they acknowledged that being involved in a cohort of nurses specifically from within the forensic setting was a benefit to the programme, due to their shared understanding, appreciation, and experience of the forensic setting. This led to the acknowledgement that a more mixed cohort may not have had the same impact.” (9)(p516)  “Delivery of MSCR content in a group format offered opportunity to reflect and learn from each other. Sharing with peers tended to normalize experiences of stress and reduce isolation.” (28)(p614)  “From the perspective of senior nurses, there was some reluctance to divulge work problems and emotional difficulties in the presence of junior staff.” (28)(p614) |
| **Finding RQ3a-5: Consolidating learning and sustaining knowledge and skills acquired** related to feedback, reminders, refresher sessions and role play. | | (11,22,28,29) | Studies described several ways to help enhance and/or consolidate new knowledge and skills. Participants in a few studies suggested that refresher sessions would be beneficial to maintain knowledge and skills (11,22,28). The benefit of receiving reminder emails with information about underpinning principles of the intervention was noted by participants in one study (29). Further, in another study, workshops were valued as an opportunity to enhance knowledge and practice new skills, specifically during role play exercises (22). | | “In regard to feasibility of the MSCR program in the longer term, there were a few suggested modifications that included… adding opportunities for informal follow-up sessions.” (28)(p613)  “They felt that ‘refresher’ sessions could provide an opportunity to ‘try out’ different strategies and maintain their motivation to  apply the principles in their day-to-day work” (11)(p838) |
| **Implementation strategy** | | | | | |
| **Finding RQ3a-6: Management/leadership buy-in and support at all levels** related to prioritisation, proactivity, and promotion of the intervention, appropriate authority and continuous commitment, appropriate authority and demonstrating the value placed on it. | | (3,11,14,17,20,22,23,26,27) | Several studies reported the importance of leadership. Facilitators included leadership to ensure ‘buy-in’ to the intervention specifically at a senior and executive level (11,22) with leadership collaboration at every level of the organisation, (11,17) are also recognised as important. One study found that, leaders bought into the intervention due to an existing evidence base and used said evidence base to obtain funding to implement the intervention (17). Two studies highlighted that an engaged first-line leader needs capabilities to engage employees (11,14). Support from management was highlighted in two studies (11,26).  Conversely, when leadership did not foster buy-in (11), were not proactive (22), or supportive (23), and did not prioritise the implementation intervention highly enough (3,20), were reported as barriers. Additionally, those who supported the intervention at the outset but did not demonstrated commitment throughout the implementation, (27) were reported as barriers. One study highlighted that managers who lack the authority to make decisions and act, e.g., due to organisational management structures, also hindered implementation (20). | | "Some recognised the importance of brokering executive-level buy-in and commitment as well as fostering their ongoing engagement and support to help facilitate study delivery" (22)(p 60)  “An important facilitating factor in the inner setting domain was an engaged first line manager, who also communicated clearly about the importance of everyone´s engagement.” (14)(pp12-13)  "Directors identified barriers to the implementation process including insufficient planning for an effective buy-in pitch to staff regarding the benefits of mindfulness and to familiarize them with the mindfulness expert." (17)(p866) |
| **Finding RQ3a-7. Communication of clear, succinct, relevant, timely information** related to the use of formal, appropriate, and varied channels, to share information both vertically and horizontally at every stage of the implementation. | | (3,9,11,14,17,22,24,26,27) | The importance of effective communication as a facilitator to implementation was frequently reported. Several studies deemed it important to establish formal, appropriate, and varied communication channels (e.g., using intranet, payslips, posters, face-to-face) (9,14,22,26,27), not just relying on emails, for example (9,22). Specifically, communication needed to be timely (9) and in a format that was succinct and practical (22).  One study found clear communication enabled employee engagement in the intervention (14). Likewise, well established employee engagement was a factor that facilitated communication recruitment, implementation, and evaluation of the intervention (11). A few studies suggested the use of existing communication structures (17,22,24); whilst another highlighted the need to establish these where they did not exist (11).  One study reported promoting vertical and horizontal communication (17); and working in a collaborative manner with HR (22) were facilitators whereas lack of communication across groups of employees was highlighted as a barrier in another studies (14).  In terms of the timing of communication a few studies reported that regular check-ins at every stage of implementation (e.g., development, evaluation, progress, and feedback) was an important facilitator (9,26,27).  One study found a barrier to implementation was a lack of an effective buy-in pitch to staff regarding the benefits of the intervention (17). Two studies found participants of the intervention were unclear about the goal of the project (3,24) and a lack of awareness of what support was available (3). | | “Members from each level of the organizational hierarchy credited one of the clinical managers and the mindfulness expert as being instrumental as agents of change throughout the organizational structure, networking vertically to address leadership needs and horizontally to address staff needs.” (17)(p868)  “Despite extensive staff information and communication efforts prior to study start, some ED providers felt not well informed about the project purpose and process.” (24)(p4) |
| **Finding RQ3a-8: Change agents** related to a range of individuals, both internal and external who could drive or championed implementation. | | (17,22,26,27) | A few studies highlighted the importance of individuals or groups of individuals taking on the role as change agents to promote, lead or support the implement the intervention. These individuals could be senior managers, (22) clinical managers, topic experts (17) or inspirational colleagues (26). Additionally, collegiate support across mentors was believed to be a facilitator (22). | | "Successful implementation of an employee’s RTW (return to work) plan was more likely to succeed when senior management were engaged in the process and recognised as key agents to lead and promote a healthy workplace culture" (22)(p67) |
| **Finding RQ3a-9: Assistance and backing to engage in the intervention** related to support from colleagues, managers, intervention experts and technology teams for both employee-level participation and for the intervention activities themselves. | | (3,9,11,14,22,23,26,27,29) | Practical support was a prominent issue. A few studies highlighted that management and colleagues support facilitated attendance at training and other events associated with the intervention (23,26). Conversely, several studies suggested a lack of time (22,27) or lack of support across a range of issues, for example, technical training (14,27,29), support for working groups (3); additional information and training (9) and ongoing engagement and support (22). | | “Whilst some mentors felt well prepared for their involvement in the programme, others would have liked additional, timelier, information and training“ (9)(p997)  “Barriers to use of the strategy were: insufficient support  for use of the strategy from team coaches, insufficient support from  higher layers of management and HR, lack of time, educational level of DPPs, lack of computer skills in team members"  (27)(p488) |
| **Finding RQ3a-10: Stakeholder engagement** related to collaborative working, involvement in decisions and participator approaches to implementation involving key stakeholders. | | (14,22,24,26,27) | Many studies reported that stakeholder engagement was a facilitator to successful implementation. This included engaging staff in, for example, buy-in to the proposed intervention (24), contact between different stakeholders to discuss the implementation strategy (27), decision making about current issues and concerns (22), decision-making using a participatory approach to using consensus approach to address key issues (14), and suggesting solutions (26). One study noted however that the size of the group could be a barrier, with bigger groups being more difficult to engage (14). | | “In these guided health circles, participants collectively analyzed and discussed potential solutions for work problems and defined an action plan with concrete measures, persons responsible, and deadlines.” (24)(p4)  “Engaging staff in the decision-making process allows them jointly to identify current issues and concerns and to agree on what type of support is required from OH.” (22)(p61) |
| **FindingRQ3a-11: Clarity of roles, responsibilities and boundaries** relate to local implementation and intervention providers**.** | | (3,9,22) | Establishing clearly defined roles and responsibilities within Occupational Health with regards to local implementation was deemed paramount to study success (22). Likewise, another study highlighted the importance of clearly defines roles for mentor and mentees (9). One study reported lack of clarity about roles and responsibilities was also a barrier to implementation (3). | | “In terms of resource implications associated with delivering the intervention, it was identified that establishing clearly defined roles and responsibilities within OH teams with regards to local implementation of the study is paramount to study success.” (22)(p67) |
| **Context** | | | | |  |
| **FindingRQ3a-12: Internal resources - Time** related to time to plan, integrate, and engage in implementation and deliver of the intervention. | | (3,9,14,17,20,22–24,26,27,29,30) | In many studies, participants reported time was as a barrier to engaging with the intervention (3,9,14,17,20,22–24,26,27,29,30). Several underlying reasons were provided. For example, staff shortages (14,20,24,26,27), staff turnover (14,27), unscheduled absences (20), high workloads (3,20,26,27,30), competing priorities and projects (3), and shift patterns (9,23,30), all contributed to a lack of time to engage with the intervention.  Participants in some studies said that the intervention added more tasks or time to their already busy schedules (17,22) making it difficult to find time for the intervention. Additionally, it was reported that taking time to complete interventions outside of work hours contradicted the goal of the intervention itself, i.e., to improve work-life balance (9), or that distractions and demands in participants personal lives hindered participants’ opportunity participate with the intervention (29).  Some studies also highlighted that preparing resources facilitated implementation (14,26). Alternatively, a lack of preparation, e.g., not planning staff coverage for training sessions and opportunities to use the intervention (17), not arranging resources to deliver the intervention (22), and not allowing participants time to transition between the intervention (which can evoke strong emotions) and work tasks (30), were identified implementation barriers. | | "The primary barrier [to staff buy-in] identified by direct care providers was lack of planning to provide coverage for them to attend training sessions. Several participants in our focus groups who wanted to participate were unable to for this reason." (17)(p866)  "In the interviews (with team coaches, HR professional, and nurse), two general contextual factors were mentioned. Firstly, due to a shortage of health care personnel on the labour market, too many vacancies existed within the teams. This resulted in high workload for the team members, preventing them from participating in the strategy." (27)(p486)  “A few of the focus group participants highlighted how they had struggled to get the time to attend the Rounds regularly because of their shift system making it difficult to take a full lunch break. Many felt too busy and felt the demand of getting back to the ward.“ (30)(p948) |
| **Finding RQ3a-13: Internal resources - Physical environment** related to appropriate and accessible intervention location. | | (9,17,23,25,26,30) | A few studies highlighted the importance of the physical environment (26), which if inappropriate could be a barrier to attendance or not conducive with the interventions. In terms of location attendance was a barrier if the intervention was not at participants main base (23,30) or if the bases for mentor and mentee differed (9). Suitability related to the intervention venue size and privacy e.g. too small, ‘noisy’ and ‘disruptive’ (17,25). | | “Training sessions were held on-site in hospital conference rooms to facilitate participation. Some participants cited this as convenient, but others cited it as a barrier because the space was too small for some of the group mindfulness and yoga practices that required participants to lie on the floor and because the location could be “noisy” and “disruptive’.“ (17)(p867) |
| **Mental models** | | | | |  |
| **Finding RQ3a-14: Previous experience of mental health interventions and training** related to positive or negative experience of intervention or training | | (17,23) | Implementation of the intervention was facilitated if participants had a previous positive experience with the intervention or training (17). Conversely, a negative experience with training could hinder implementation (23). | | ‘Past experience with mindfulness was viewed as positively influencing attitudes toward the initiative and thus facilitating implementation.’ (17)(p865) |
| **Finding RQ3a-15: Perception of intervention as motivation to engage** related to participants’relatability and level of interest in workplace stress, perceptions of the usefulness and positive reputation, of the intervention and progress made. | | (3,11,14,17,22–24,26–29) | Two studies reported that the implementation of the intervention was facilitated if participants were motivated to address workplace stress (27) and improve workplace conditions (24).  Participant’s perception of the intervention could act as facilitators to its implementation. Participants who viewed the intervention positively (17)*;* or perceived its value (23,26,28,29) were motivated to participate in the intervention. Another study recognised that time was required to establish a positive reputation for the intervention (11).  Encouragement and positive feedback about intervention engagement from co-workers acted as a facilitator (26). Likewise, if participants perceived progress this boosted their motivation to engage in the intervention (14,26,28). However, motivation decreased as progress reached a plateau (14).  A barrier to the intervention implementation was present if the intervention was perceived to be like existing practice (22,26). Likewise, if parts of the intervention are not perceived as relevant, engagement decreases (3). Additionally, one study reported barriers to implementation existed where participants perceived that the intervention did not addressing route cause of stress or burnout (e.g., staff shortages or excessive workload) (23). | | “This growing sense that they functioned more effectively tended to persuade nurses of the value of MSCR practices.” (28)(p613)  “noticing progress boosted their willingness to continue. After a while, however, when they noticed that the results were similar from week to week, some felt they had lost the impetus to give regular feedback“ (14)(p13) |
| **Finding RQ3a-16: Stigma about mental health and perceived confidentiality issues** related to participants willingness to engage with the intervention, be open and talk about their experiences or gain insight into mental health problems. | | (9,11,23,25,27,28,30) | Several studies reported a perception that being open about mental health and work stress was associated with stigmatisation.  One study noted the importance of feeling safe and free from negative repercussions, criticism, or blame (30). One study reported some participants perceived that those who attended the intervention were unable to cope with stress (23). Another study reported that similar perceptions had initially inhibited those who had attended the intervention which they believed might be associated with a lack of resilience. However, these perceptions shifted following attendance (9). Likewise, another study reported that attending the intervention had destigmatised post-traumatic stress reaction (25).  A few studies reported that sharing subjective experiences in a safe way to create dialogue about mental health had served to combat stigma (11) and had been a relief and ‘de-shaming’ (30). Another reported attendance had meant participants of the intervention were now comfortable devoting resources to their own well-being (28).  Additionally, the ability of staff to be open about work stress was perceived as a barrier to engagement in the implementation strategy and as it was perceived it could have detrimental consequence (27). | | "The personal stories reportedly increased their insight into what colleagues may be experiencing and also helped to combat the  stigma associated with the condition. This process challenged their preconceived ideas about mental illness and had a lasting emotional impact on them" (11)(p838).  "One notable difference was that the Beyond Silence participants talked about the significant impact of leaders who were able to share their personal experiences in an honest and authentic way. They  noted the leaders’ ability to use these personal experiences to create an open dialogue about mental health issues in a supportive, non-judgmental way and that their advice had more impact since “they’ve been there.” (11)(p838)  "there was resistance to the strategy. On the one hand, there was a lack of awareness and readiness for change in team members. On the other hand, team members resisted the strategy because they felt they had not been involved in the decision-making process of participation in the strategy, and because they were afraid of detrimental consequences (such as being reprimanded for speaking up) if they openly talked about work stress" (27)(p488) |
| **Summary of evidence provided for RQ3b SMEs** | | | | | |
|  | | **Intervention Activities** | | | |
| **‘Code’ and Name** | | **Studies contributing to review findings** | **Narrative Description** | **Example quotes** | **SECTOR SPECIFIC EVIDENCE** |
| **RQ3b-1: Intervention content** related to relevance and tailoring to the needs of the organisation or sector context and participants, varied and easy to use. | | (11,31–33) | Clear and simple intervention components and designs were seen facilitative to implementing interventions (11,31–33).  Regarding formatting, interventions should be easy to navigate, access and monitor progress, particularly for digital interventions (32), activities should be short in length (32,33), and a variety should be offered in terms of delivery mode (33), content and types of activities were also valued (11,33).  With regards to content, materials designed to address specific needs of organisations (Moll, et al., 2018) and particularly sectors and participants (Ross, et al., 2019) were valued by employees. | “Many participants described shorter-length practices as easier to engage with than longer practices.” (33)(p9)  “The short exercises were seen positively” (32) (p9)  “Some participants noted that they enjoyed having a variety of content formats (ie, audio, video, and written) and types of practice (eg, loving-kindness and body scan) to try each week. The availability of different content allowed participants to find what worked best for them.” (33)(p10)  “They [connectors] also emphasised how GAT training was ‘pitched at the right level’, that is, specifically for construction workers rather than for office workers, without too much focus on psychology/mental health.” (31)(p8) | +ICT  +Construction  +Healthcare |
| **RQ3b-2 Fostering relationships and openness** related to collaboration, communication, and reflective dialogue about mental health. | | (11,31) | Good relationships between all stakeholders in the organisation with existing communication channels facilitates intervention implementation (11). Time should be allowed to develop such relationships and channels of communication prior to implementation if they do not already exist (11).  Activities that create and reinforce opportunities for open, authentic, and reflective dialogue about mental health at work with peers and leaders were valued by employees (11,31). Support for peer intervention providers was also highlighted as important (31). | In the first organization, there was a history of collaboration with leaders in Health Safety and Wellness, support from senior leadership, and an advisory team of union representatives, managers, and front-line workers from both clinical and nonclinical areas. This level of engagement facilitated communication for recruitment, implementation and evaluation… In the second organization, time was needed to establish new relationships, channels of communication…” (11) (p838)  “Participants talked about how much they valued the opportunity to reflect on and discuss issues and scenarios that were directly relevant to their day-to-day work” (11) (p838)  “They described how through the MATES program, talking about suicide had started to become acceptable, whereas in the past, this was seen as taboo.” (31)(p7) | +Construction  +Healthcare |
|  | | **Implementation Strategies** | | | |
| **RQ3b-3: Management /leadership buy-in and support** related to promoting and engaging employees in intervention. | | (11,32,33) | Leadership commitment to support intervention activities actively and continuously was facilitative. Leadership ‘buy-in’, i.e. from top management and local management (32), was seen as important to engage employees and create systematic change (11), for example, management sharing resources and communicating information about the intervention, attending intervention sessions, designating time for employees to complete the intervention with attention to scheduling (32,33) and aligning intervention activities with those already in the organisation. A lack of leadership buy-in to support these important tasks was reported as a barrier (11,32,33). | "the top management made the final decisions about participating in this study and that employees could participate in kick-off meetings during working hours, but the app use had to take place on their own time. Although the HR managers were a rich source of information and a suitable first point of contact in the companies, more effort should have been taken to involve the top management because they made the strategic decisions." (32) (p6)  "Although some participants were proactive in sharing resources and information with their team, others found it more difficult and discussed the importance of leadership “buy-in” for real change to occur. One participant, for example, noted that she had changed but that broader system change is still needed." (11) (p839) | +Healthcare  +ICT |
| **RQ3b-4: Promoting participation in the intervention** related to multiple promotion strategies to increase local visibility through change agents and managers. | | (31,32) | Multiple intervention promotion strategies were beneficial, as some work better than others (32). Additional helpful strategies, from within the organisation promoting intervention visibility included regular promotion of the intervention and personal communication and commitment from change agents and managers to increase intervention awareness, implementation success and motivation participants to engage in the intervention (31,32). | “The paper brochures did not reach many employees, and a message from the HR and the management was mentioned to have more impact on participation." (32) (p8)  “Connectors stressed that MATES’s high visibility on sites, as well as their passion and engagement with workers was integral to the success of MATES.” (31) (p9) | +ICT  +Construction |
| **RQ3b-5: Clarity of roles, responsibilities, and boundaries** relate to local implementation and intervention providers. | | (31,32) | Agreement and clarity of roles in relation to implementation is important. This may include identifying who is responsible for monitoring and promoting intervention engagement (32), distinguishing to what extent are leaders involved in supporting the intervention implementation (32), and defining roles of intervention providers to set expectations and set boundaries (31). A lack of role clarity early on can create barriers to implementation (32). | “Participants stressed another effective aspect of the MATES model was the clearly defined roles for volunteers, emphasising that they are not mental health workers nor there to ‘fix’ problems. Rather, MATES training provides the skills to recognise when someone needs help, and to be able to connect the person to assistance” (31) (p8) | +ICT  +Construction |
|  | | **Context** | | | |
| **RQ3b-6: Physical environment** related to appropriate and accessible intervention location. | | (33) | Workplace contextual factors, such as frequent interruptions, noise levels, shared workspaces and busy work conditions, were considered barriers to participant engagement with the intervention, particularly for guided audio intervention components (33). | "Frequent interruptions, noise levels, shared workspaces, and busy working conditions were often cited" as barriers to engagement with the intervention, particularly the guided audio content.” (33) (p9) | Healthcare |
|  | | **Mental Models** | | | |
| **RQ3b-7 Perception of intervention as motivation to engage** related to participants’ relatability and level of interest in workplace stress, positive reputation of the intervention, perceptions of the usefulness, and progress | | (11,31–34) | Participant beliefs that the intervention is relatable was a key motivator for engagement (11,31–34), e.g., that the intervention fits with their day to day work environment, their life, their own abilities (31,33), and was of benefit to them (32,33).  Incongruence with day-to-day working lives or a perceived lack of benefit (11,32,33) were barriers.  Sector relevant examples, case scenarios, leaders sharing personal experiences and presenting sector specific statistics about suicide rates or mental health were seen as a good way to ensure relatability, as well as reduce stigma-related barriers to engaging in mental health interventions (11,31).  Recent experiences of stressful life events or stressful work experiences were considered motivating factors for top management/owners to engage with mental health activities for themselves and to take a proactive management strategy for workplace mental health (34).  Conversely, some personal factors, e.g., substance misuse (31), introversion (11), being in a stressful state (33), and having a stoic attitude (31) can still act as barriers to help-seeking of engaging for a minority of people.  Participant positive beliefs about the credibility of the intervention increased support for the intervention and motivation for engagement (31). Building a positive reputation before implementation facilitates implementation (e.g. by management endorsing and communicating the intervention in a positive light) (11).  A perceived lack of credibility was a barrier (32). | “[connectors said that] learning about high construction industry suicide rates was a motivator to continue training” (31) (p7)  “several interviewees spoke of how they were able to relate to stories from MATES delegates and male peers about help-seeking experiences, which enabled them to move past traditional barriers to reach out for help” (31) (p9)  “…aligning the intervention activities with existing activities in the company makes the process simpler for the employee…” (32) (p8)  “Clients reported that MATES workers understand the problems that are unique to those working in the construction industry and that they ‘speak the same language’.” (31) (p9)  “They noted the leaders’ ability to use these personal experiences to create an open dialogue about mental health issues in a supportive, non-judgmental way and that their advice had more impact since “they’ve been there.” (11) (p838)  "the credibility of the videos was questioned and it was difficult to find time to watch them." (32) (p9)  “In the second organization, time was needed to establish…a positive reputation for the educational initiatives." (11) (p838) | +Healthcare  +Construction  +ICT |

**References**

1. Bourbonnais R, Jauvin N, Dussault J, Vézina M. Evaluation of an intervention to prevent mental health problems among correctional officers. Improv Organ Interv Stress Well- Addressing Process Context. 2012;187–215.

2. Schelvis R, Wiezer N, Blatter B, van Genabeek J, Hengel K, Bohlmeijer E, et al. Evaluating the implementation process of a participatory organizational level occupational health intervention in schools. BMC PUBLIC Health. 2016;16.

3. Aust B, Rugulies R, Finken A, Jensen C. When workplace interventions lead to negative effects: Learning from failures. Scand J Public Health. 2010 Feb;38(3_suppl):106–19.

4. Jenny GJ, Brauchli R, Inauen A, Füllemann D, Fridrich A, Bauer GF. Process and outcome evaluation of an organizational level stress management intervention in Switzerland. Health Promot Int. 2015;30(3):573–85.

5. Mellor N, Smith P, Mackay C, Palferman D, Mellor N, Smith P, et al. The ‘Management Standards’ for stress in large organizations. Int J Workplace Health Manag. 2013;6(1):4–17.

6. Donaldson-Feilder E, Lewis R, Yarker J. Preventing Stress: Promoting Positive Manager Behaviour [Internet]. Chartered Institute of Personnel and Development; 2011 [cited 2022 Sep 12]. Available from: https://www.cipd.co.uk/Images/preventing-stress_2011-phase-4-organisations-implement-in-practice_tcm18-10572.pdf

7. LaMontagne AD, Martin A, Page K, Reavley N, Noblet A, Milner A, et al. An integrated workplace mental health intervention in Victoria Police: results of a cluster-randomised trial [Internet]. Australia: Centre for Population Health Research, Deakin University; 2017 [cited 2022 Dec 12] p. 63. Report No.: 052.1-0317-R04. Available from: https://research.iscrr.com.au/__data/assets/pdf_file/0020/1024715/an-integrated-workplace-mental-health-intervention-in-Victoria-Police.pdf

8. Carolan S, de Visser R, Carolan S, de Visser RO. Employees’ Perspectives on the Facilitators and Barriers to Engaging With Digital Mental Health Interventions in the Workplace: Qualitative Study. JMIR Ment Health. 2018;5(1).

9. Davey Z, Jackson D, Henshall C. The value of nurse mentoring relationships: Lessons learnt from a work‐based resilience enhancement programme for nurses working in the forensic setting. Int J Ment Health Nurs. 2020 Oct;29(5):992–1001.

10. Evans R, Bell S, Brockman R, Campbell R, Copeland L, Fisher H, et al. Wellbeing in Secondary Education (WISE) Study to Improve the Mental Health and Wellbeing of Teachers: A Complex System Approach to Understanding Intervention Acceptability. Prev Sci. 2022 Aug;23(6):922–33.

11. Moll SE, VandenBussche J, Brooks K, Kirsh B, Stuart H, Patten S, et al. Workplace Mental Health Training in Health Care: Key Ingredients of Implementation. Can J Psychiatry. 2018 Dec 1;63(12):834–41.

12. Narayanasamy M, Geraghty J, Coole C, Nouri F, Thomson L, Callaghan P, et al. Mental Health First Aid in the Workplace: A Feasibility Study [Internet]. Wigston: IOSH; 2018. Available from: https://iosh.com/media/3722/mhfa-at-work-full-report.pdf

13. Montero-Marin J, Kuyken W, Gasion V, Barcelo-Soler A, Rojas L, Manrique A, et al. Feasibility and Effectiveness of a Workplace-Adapted Mindfulness-Based Programme to Reduce Stress in Workers at a Private Sector Logistics Company: An Exploratory Mixed Methods Study. Int J Environ Res Public Health. 2020;17(5).

14. Arapovic-Johansson B, Jensen I, Wåhlin C, Björklund C, Kwak L. Process Evaluation of a Participative Organizational Intervention as a Stress Preventive Intervention for Employees in Swedish Primary Health Care. Int J Environ Res Public Health [Internet]. 2020 [cited 2021 Mar 19];17(19). Available from: https://journals.scholarsportal.info/details/16617827/v17i0019/nfp_peoapoeisphc.xml

15. Saksvik PØ, Olaniyan OS, Lysklett K, Lien M, Bjerke L. A process evaluation of a salutogenic intervention. Scand Psychol. 2015;2.

16. Mellor N, Mackay C, Packham C, Jones R, Palferman D, Webster S, et al. ‘Management Standards’ and work-related stress in Great Britain: Progress on their implementation. Saf Sci. 2011;49(7):1040–6.

17. Byron G, Ziedonis DM, McGrath C, Frazier JA, deTorrijos F, Fulwiler C. Implementation of mindfulness training for mental health staff: Organizational context and stakeholder perspectives. Mindfulness. 2015;6(4):861–72.

18. Knaak S, Luong D, McLean R, Szeto A, Dobson KS. Implementation, Uptake, and Culture Change: Results of a Key Informant Study of a Workplace Mental Health Training Program in Police Organizations in Canada. Can J Psychiatry. 2019;64(1):30S-38S.

19. Bovopoulos N, LaMontagne AD, Martin A, Jorm A. Exploring the role of mental health first aid officers in workplaces: A qualitative study using case study methodology. Int J Workplace Health Manag. 2018 Jan 1;11(6):366–81.

20. Tsarouha E, Stuber F, Seifried-Dubon T, Radionova N, Schnalzer S, Nikendei C, et al. Reflection on leadership behavior: potentials and limits in the implementation of stress-preventive leadership of middle management in hospitals - a qualitative evaluation of a participatory developed intervention. J Occup Med Toxicol. 2021 Nov 29;16(1).

21. Taylor WC, Horan A, Pinion Jr C, Liehr P. Evaluation of booster breaks in the workplace. J Occup Environ Med. 2014;56(5):529–34.

22. Parsons V, Juszczyk D, Gilsworth G, Ntani G, McCrone P, Hatch S, et al. A case management occupational health model to facilitate earlier return to work of NHS staff with common mental health disorders: a feasibility study [Internet]. 2021. Available from: DOI 10.3310/hta25120

23. Günüşen NP, Ustün B. Turkish nurses’ perspectives on a programme to reduce burnout. Int Nurs Rev. 2009 Jun;56(2):237–42.

24. Schneider A, Wehler M, Weigl M. Effects of work conditions on provider mental well-being and quality of care: a mixed-methods intervention study in the emergency department. BMC Emerg Med. 2019;19(1):1.

25. Tessier M, Lamothe J, Geoffrion S. Psychological First Aid Intervention after Exposure to a Traumatic Event at Work among Emergency Medical Services Workers. Ann WORK Expo Health. 2022 Aug 7;66(7):946–59.

26. Ejlertsson L, Heijbel B, Andersson IH, Troein M, Brorsson A. Strengthened workplace relationships facilitate recovery at work – qualitative experiences of an intervention among employees in primary health care. BMC Fam Pract [Internet]. 2021;22(1). Available from: https://www.scopus.com/inward/record.uri?eid=2-s2.0-85102242771&doi=10.1186%2fs12875-021-01388-x&partnerID=40&md5=e490cc933ec633f1b3e1cb431f54c690

27. Havermans BM, Boot CRL, Brouwers EPM, Houtman ILD, Anema JR, van der Beek AJ. Process Evaluation of a Digital Platform-Based Implementation Strategy Aimed at Work Stress Prevention in a Health Care Organization: J Occup Environ Med. 2018 Sep;60(9):e484–91.

28. Slatyer S, Craigie M, Rees C, Davis S, Dolan T, Hegney D. Nurse experience of participation in a mindfulness-based self-care and resiliency intervention. Mindfulness. 2018;9(2):610–7.

29. Lehto RH, Heeter C, Allbritton M, Wiseman M. Hospice and Palliative Care Provider Experiences With Meditation Using Mobile Applications. Oncol Nurs Forum. 2018;45(3):380–8.

30. Allen D, Spencer G, McEwan K, Catarino F, Evans R, Crooks S, et al. The Schwartz Centre Rounds: Supporting mental health workers with the emotional impact of their work. Int J Ment Health Nurs. 2020 Oct;29(5):942–52.

31. Ross V, Caton N, Gullestrup J, Kõlves K. Understanding the Barriers and Pathways to Male Help-Seeking and Help-Offering: A Mixed Methods Study of the Impact of the Mates in Construction Program. Int J Environ Res Public Health [Internet]. 2019;16(16). Available from: https://www.ncbi.nlm.nih.gov/pubmed/31430939

32. Muuraiskangas S, Harjumaa M, Kaipainen K, Ermes M, Muuraiskangas S, Harjumaa M, et al. Process and Effects Evaluation of a Digital Mental Health Intervention Targeted at Improving Occupational Well-Being: Lessons From an Intervention Study With Failed Adoption. JMIR Ment Health. 2016;3(2).

33. Kerr D, Ornelas I, Lilly M, Calhoun R, Meischke H, Kerr DC, et al. Participant Engagement in and Perspectives on a Web-Based Mindfulness Intervention for 9-1-1 Telecommunicators: Multimethod Study. J Med INTERNET Res. 2019;21(6).

34. Dawkins S, Martin A, Kilpatrick M, Scott J, Dawkins S, Martin A, et al. Reasons for Engagement SME Owner-Manager Motivations for Engaging in a Workplace Mental Health and Wellbeing Intervention. J Occup Environ Med. 2018;60(10):917–27.
